# Supplementary material for: A triple increase in global river basins with water scarcity due to future pollution
Source: Nat Commun. 2024 Feb 6;15:880. doi: 10.1038/s41467-024-44947-3 (PMC10847517; doi:10.1038/s41467-024-44947-3)
Supplement: Supplementary file 1 — Supplementary Information [file 41467_2024_44947_MOESM1_ESM.pdf]

Supporting Information

A triple increase in global river basins with water scarcity due to future pollution

Mengru Wang[1,#], Benjamin Leon Bodirsky[2], Rhode Rijneveld[1], Felicitas Beier[2,3], Mirjam P. Bak[1], Masooma Batool[4], Bram Droppers[5], Alexander Popp[2], Michelle T.H. van Vliet[5], Maryna Strokal[1]

[1] Earth Systems and Global Change Group, Wageningen University & Research, Droevendaalsesteeg 3, 6708 PB Wageningen, The Netherlands

[2] Potsdam Institute for Climate Impact Research (PIK), Leibniz Association, Telegrafenberg A56, 14412 Potsdam, Germany

[3] Humboldt University, Thaer-Institute of Agricultural and Horticultural Sciences, Invalidenstr. 42, 10099 Berlin, Germany

[4] UFZ-Helmholtz Centre for Environmental Research, Department of Computational Hydrosystems, Leipzig, Germany

[5] Department of Physical Geography, Utrecht University, PO Box 80.115, 3508 TC Utrecht, the Netherlands

#Corresponding author (Email: mengru.wang@wur.nl)

This file includes information on:

Data for clean-water scarcity assessment 2
MARINA-Nutrients Global 1.0 4
MAgPIE 9
Results of clean water scarcity assessment 13
References 39

Data for clean-water scarcity assessment

Table S1 Overview of data requirements and availability for clean-water scarcity assessment. The variables are used in equations 1 and 2 in the main text.

Variables	Description	Unit	Future data availability	Source
Q_{nat}	Natural river discharge	km^3/year	Yes	VIC-4[i] (van Vliet et al., 2016) (Li et al., 2022)
Q_{act}	Actual river discharge	km^3/year	Yes	This study following the approach described in Table S2
D_j	Water withdrawals for sector j	km^3/year	No, scenario-based	VIC-5[i] (Droppers et al., 2020) for 2010; this study for 2050, following the approach described in Table S2
EFR	Environmental flow requirements	km^3/year	Yes	This study following approach of (Pastor et al., 2014)
L	N load	Kg/year	Yes	MARINA-Nutrients Global-1.0[ii] (developed in this study, taking inputs from MAgPIE[iii] and VIC[i], a detailed description in available in the next section of this file)
C_{max}	Maximum water quality threshold of nitrogen concentrations for ecosystems	mg/L	No, assumption that it is similar to current thresholds	De Vries et al. (2013; Yu et al. (2019

[i] VIC - Variable Infiltration Capacity model;
[ii] MARINA - Model to Assess River Inputs of pollutaNts to seAs
[iii] MAgPIE - Model of Agricultural Production and its Impact on the Environment

Water availability (natural and actual river discharges) (Q_{nat} and Q_{act})

The cumulative natural river discharge at the sub-basin outlets of the rivers, i.e., river discharge before water is withdrawn for consumption, for both past (2010) and future (2050) was provided by the VIC (Variable Infiltration Capacity) model runs (van Vliet et al., 2016). The cumulative actual river discharge at the sub-basin outlets of the rivers, i.e., discharge after water is withdrawn for consumption, for both past (2010) and future (2050) was derived from the MARINA-Nutrients-Global-1.0 (Model to Assess River Inputs of pollutaNts to seAs) model that is developed in this study (see Figure S2 and Table S2 for details).

Sectoral water withdrawals (D_j)

The VIC-5 model provides sectoral (domestic, industrial, livestock, and irrigation) water withdrawals from 1979-2016 (Droppers et al., 2020). The model runs at daily time steps and executes simulations on a 0.5 ° by 0.5 ° grid scale. To average climate extremes, water withdrawals for 2010 were obtained based on the mean of yearly sectoral withdrawals from 2005-2015. The gridded data were aggregated (summed) to annual withdrawal data on a sub-basin scale. However, six small sub-basins could not be defined by a grid cell, and they fell out of the analysis. Two of these sub-basins are in North America and four in Asia. All these sub-basins have very small water withdrawals according to the MARINA-Global model (maximum 0.08 km^3/year). Since these sub-basins have little water withdrawals and are relatively small (ranging from 420 to 3077 km^2), their omission should not notably influence water scarcity values.

Future sectoral water withdrawals (*D_j*)

The VIC-5 model does not provide future withdrawal projections. Water withdrawals for 2050 were thus derived based on the changes in water withdrawals between 2010 and 2050 from the MARINA-Nutrients-Global-1.0 model developed in this study and water withdrawals in 2010 from VIC-5 as derived above. Changes in water withdrawals between 2010 and 2050 (future withdrawal factor) were calculated following Equation S1 by comparing the differences in natural and actual river discharges in these two years. Here, natural river discharge refers to the river discharge at the outlet of sub-basins before water is withdrawn for consumption. Actual river discharge refers to the river discharge at the outlet of sub-basins after water is withdrawn for consumption by sectors. Details on how natural and actual river discharges are derived in this study are in Table S2 category 4.

$$\text{Future withdrawal factor} = \frac{(Q_{nat}^{2050} - Q_{act}^{2050})}{(Q_{nat}^{2010} - Q_{act}^{2010})} \ (\boldsymbol{Eq\ S1})$$

The future withdrawal factor was multiplied with the 2010 sectoral withdrawals to obtain 2050 withdrawals for each sector. This method allows to assess spatial differences in total withdrawals among the sub-basins, yet the share between sectors remains similar between 2010 and 2050.

Environmental flow requirements (EFRs)

We derived the annual EFRs for sub-basins following the approach of Pastor et al. (2014) using mean annual flow (MAF) and mean monthly flow (MMF) provided by the VIC (Variable Infiltration Capacity) model runs (van Vliet et al., 2016). We first determined the hydrological seasons as follows: low-flow month when MMF ≤ 0.4·MAF, high-flow month when MMF > 0.8·MAF, intermediate-flow month when 0.4·MAF < MMF ≤ 0.8·MAF. Next, we calculated the EFRs based on the hydrological seasons: EFRs for low-flow months are estimated as 0.6·MMF, for high-flow months are 0.3·MMF, for intermediate-flow months are 0.45·MMF. Last, we aggregate the monthly EFRs (from last step) to annual EFRs as a fraction of MAF. We derived the EFRs for 2010 and 2050. Results vary between 30% and 38% among sub-basins (see Figure S9). This is comparable to the conclusion of Pastor et al. (2014) who suggested reserving an annual average of 37% of global annual flows to keep ecosystems in a fair ecological condition for global water availability assessments.

Nitrogen load (*L*)

The actual N load (kg/year) at the outlet of sub-basins was calculated by the MARINA-Nutrients-Global-1.0 model developed in this study, referred as $\mathrm{OT_{F.y.j}}$ in Equation S3 for individual rivers or tributaries, and $\mathrm{OC_{F.y.j}}$ in Equation S4 for main channel. See Figure S1 for the definition of tributary and main channel. A detailed model description is available in Section "MARINA-Nutrients-Global-1.0" of this SI, where we described the main equations of the model (Equations S2-S4), scheme for sub-basins defined in the model (Figure S1), model inputs and their sources (Figure S2 and Table S2).

Maximum water quality threshold of nitrogen concentrations for ecosystems (*C_{max}*)

For C_{max}, we took 1 TDN mg/L as the threshold for avoiding eutrophication in the aquatic ecosystem, based on the study of De Vries et al. (2013; Yu et al. (2019. These thresholds remain the same for 2010 and 2050.

MARINA-Nutrients Global 1.0

The MARINA (Model to Assess River Inputs of pollutaNts to seAs) quantifies annual river export of multiple pollutants (i.e. nitrogen (N), phosphorus, micro- and macro plastic, pathogens, chemicals) to seas from point and diffuse sources for >10,000 subbasins worldwide (Strokal et al., 2019). In this study, we developed the MARINA-Nutrients-Global-1.0 model to assess river and coastal water pollution by total dissolved N (TDN) to seas from both diffuse and point sources in 2010 and 2050. TDN includes dissolved inorganic and organic N (DIN and DON). N from diffuse sources in agriculture and non-agriculture land are based on the Model of Agricultural Production and its Impact on the Environment (MAgPIE), while N from point sources is based on a previous version of MARINA developed by Strokal et al. (2021 to quantify N inputs to global rivers from sewage systems (Figure 1 in the main text).

The overall equation of MARINA-Nutrients-Global-1.0 to quantify river export of TDN to seas is summarized in Equation S2, variables in which is further used in Equations S3 and S4 to quantify TDN loads at the sub-basin outlets.

$$\mathrm{M_{F.y.j}} = \mathrm{RS_{F.y.j}} * \mathrm{FE_{riv.F.outlet.j}} * \mathrm{FE_{riv.F.mouth.j}}\ (\boldsymbol{Eq\ S2})$$

$M_{F.y.j}$ stands for the export of nitrogen in form F (DIN, DON) to the river mouth by source y from sub-basin j. First, the model quantifies inputs of N in form F from diffuse or point source y to surface waters in sub-basin j ($RS_{F.y.j}$). The model considers diffuse sources such as synthetic fertilizers, animal manure and human excreta in agriculture, and leaching of organic matter from (non-)agricultural areas. For diffuse source, $RS_{F.y.j}$ is quantified based on N input, uptake and retention of N on land as a function of runoff. The model includes point sources such as sewage systems and open defecation. Secondly, the model quantifies the fraction of N that reaches the outlet of each sub-basin j ($FE_{riv.F.outlet.j}$). Thirdly, the model quantifies the fraction of N that is exported from sub-basin outlets to the river mouths, i.e., the point where nutrients are discharged into the sea ($FE_{riv.F.mouth.j}$). $FE_{riv.F.outlet.j}$ and $FE_{riv.F.mouth.j}$ are calculated taking into consideration of N retention in rivers through dam retention, denitrification and water consumption.

Deriving Nitrogen Load (variable *L* in Equation 2 in the main text)

To calculate the water quality-based indicator ($S_{quality}$ in Equation 2 in the main text), in this study, the pollutant load (*L* in Equation 2) is based on the load of total dissolved nitrogen (TDN) at sub-basins outlets simulated by MARINA-Nutrients. A sub-basin in the MARINA-Nutrients model can be formed by and subsequently categorized as an individual river, a tributary (T), or as a main channel (C) (see Figure S1 as an example). The N load at the outlets of individual rivers and tributaries were calculated with Equation S3:

$$\mathrm{OT_{F.y.j}} = \mathrm{RS_{F.y.j}} * \mathrm{FE_{riv.F.outlet.j}}\ (\boldsymbol{Eq\ S3})$$

$OT_{F.y.j}$ stands for the load of N in form F (DIN, DON) by source y at the outlet of sub-basin j as an individual river or tributary. $RS_{F.y.j}$ and $FE_{riv.F.outlet.j}$ are the same parameters as in Equation S2.

Main channels receive N from their own sub-basins as well as N from tributaries draining into the channels. The N loads at the outlets of the main channels were therefore calculated with Equation S4:

$$\mathrm{OC_{F.y.j}} = \mathrm{RS_{F.y.j}} * \mathrm{FE_{riv.F.outlet.j}} + \sum_{1}^{k} \mathrm{OT_{F.y.k}} * \mathrm{FE_{riv.F.channel.k.j}}\ (\boldsymbol{Eq\ S4})$$

$OC_{F.y.j}$ stands for the cumulative load of N in form F (DIN, DON) by source y at the outlet of sub-basin j as a main channel. $OT_{F.y.k}$ stands for the inputs of N by source y from tributary k to the main channel j. $FE_{riv.F.channel.k.j}$ stands for the fraction of N that is exported from tributary k to the outlet of a main channel j.

All further equations in the MARINA-Nutrients-Global-1.0 model to quantify the above mentioned variables used in Equations S2-S4 ($\mathrm{RS_{F.y.j}}, \mathrm{FE_{riv.F.outlet.j}}, \mathrm{FE_{riv.F.mouth.j}}$) are available in the Supporting Information (SI) of Wang et al. (2020b (See Boxes A1 and A2 in https://agupubs.onlinelibrary.wiley.com/action/downloadSupplement?doi=10.1029%2F2019EF001280&file=Supporting-Information_final_clean_S.PDF). $\mathrm{FE_{riv.F.channel.k.j}}$ is introduced as a new variable in this

study, the calculation of which follows the equation for calculating $FE_{riv.F.mouth.j}$. All calculated FE parameters by the model using the model inputs in Figure S2 are available in the data repository (See Data availability section in the main text).

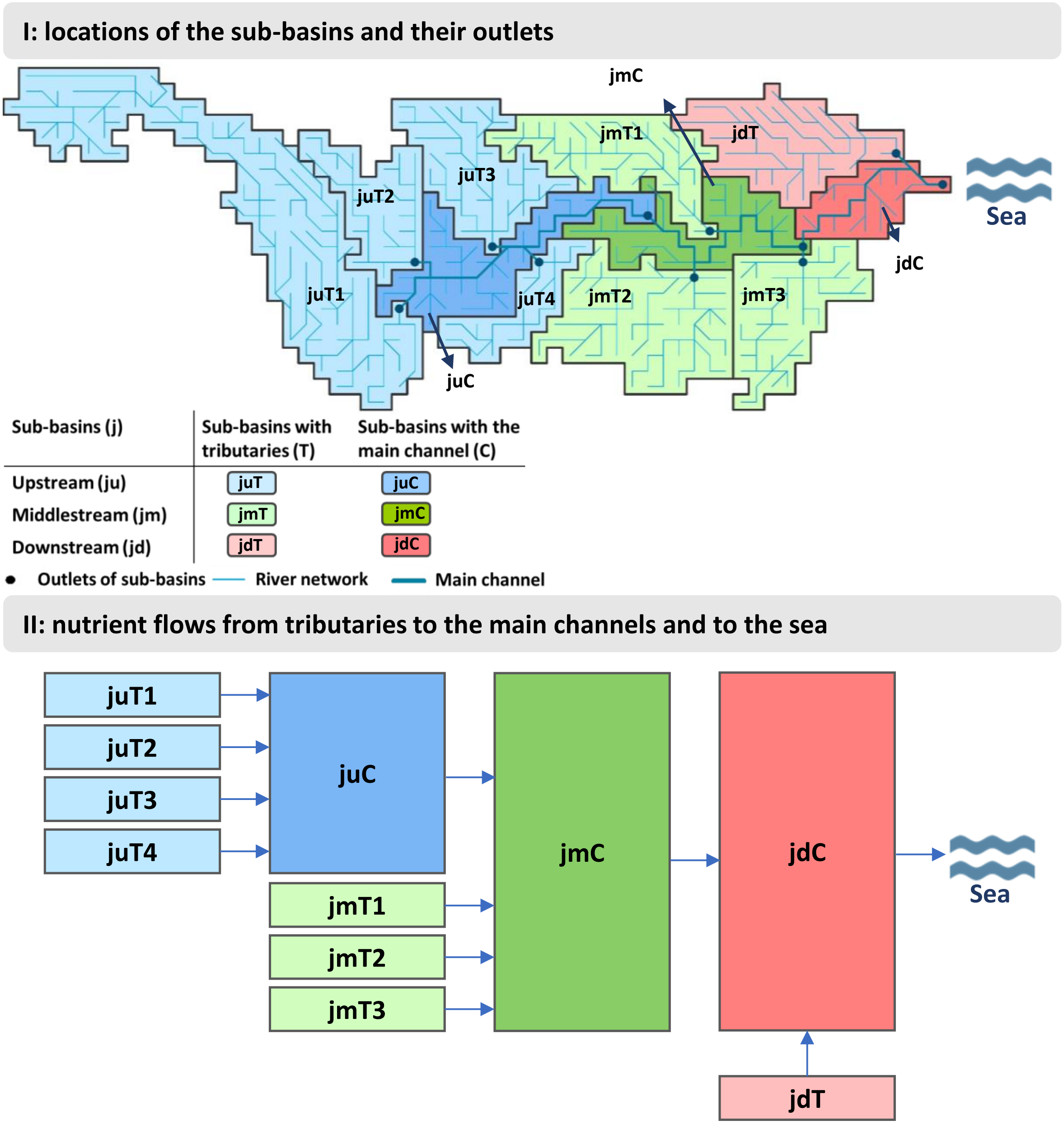

Figure S1 Scheme for nutrient flows from sub-basins to rivers and to the sea in the MARINA-Nutrients-Global-1.0-model. This figure shows the sub-basins of the Yangtze River as an example. We distinguish among upstream (u), middlestream (m) and downstream (d) sub-basins. Sub-basins are categorized as a tributary (T) or a main channel (C).

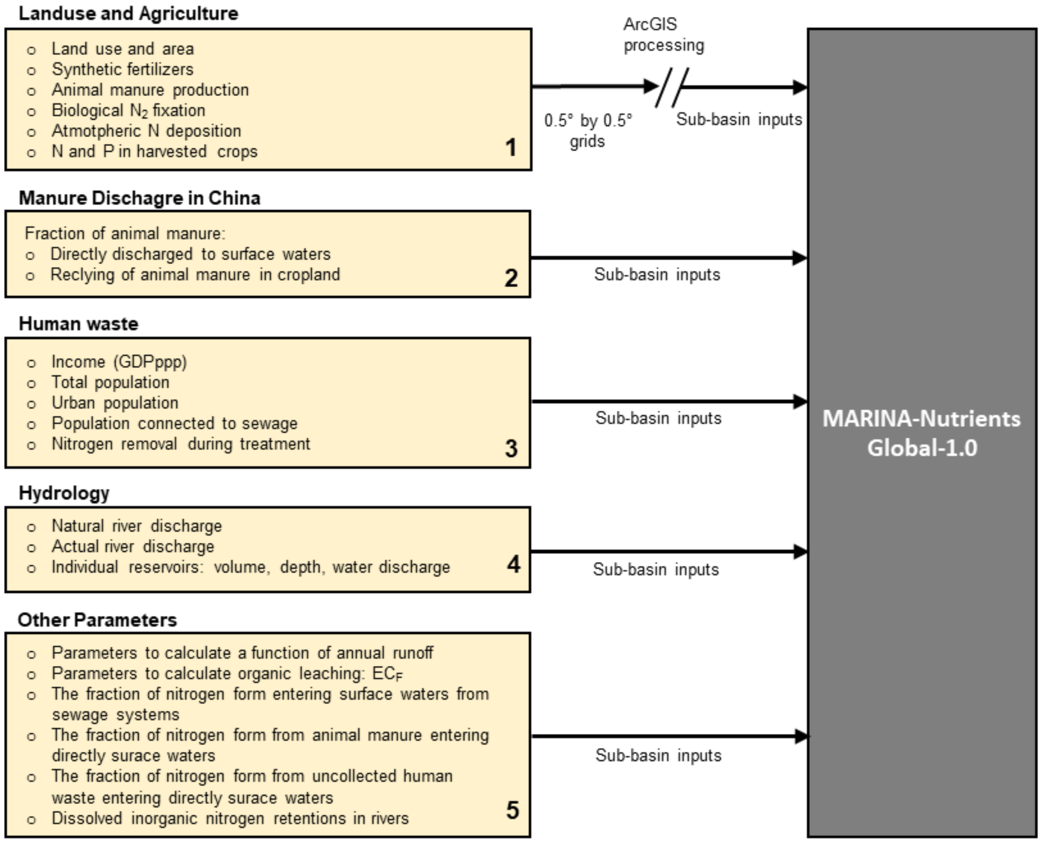

Figure S2 Model inputs for MARINA-Nutrients-Global-1.0. The sources of the databases and how the inputs were processed to be used in MARINA-Nutrients-Global-1.0 are in Table S2. The model uses region-specific manure discharge for China as indicated in the Figure. Explanation for this is available in Table S2.

Table S2 Sources of model inputs in Figure S2 and description of how model inputs were derived from their sources for being used in the MARINA-Nutrients Global 1.0.

Model input category	Description
1 Land use and agriculture	These model inputs were derived from the MAgPIE model. We aggregated MAgPIE data from 0.5° latitude by 0.5° longitude grids to sub-basins using ArcGIS. For example, the use of synthetic fertilizers in agriculture in a sub-basin is a sum of the use of synthetic fertilizer of all grids covered by the sub-basin.
2 Manure discharge in China[i]	Studies show that in 2010, China had poor manure management (Strokal et al., 2016; Wang et al., 2020a; Wang et al., 2018). Part of the collected animal manure were not treated properly or reused in cropland but were discharged to rivers directly. Thus, for the Chinese sub-basins in 2010, we took data for the direct discharge of animal manure from the MARINA-Nutrients-China-2.0. For 2050, we assume that there is no direct discharge of animal manure in China according to the recently introduced manure management regulations by Chinese government. Thus for 2050, we used the data on manure management from model input category 1.
3 Human waste	These data were taken directly from the MARINA-Multi-Global-1.0 model on the sub-basin scale for 2010 and 2050. Details of these model inputs and how they were derived from multiple databases can be found in Supplementary Table 3 of Strokal et al. (2021. All model inputs at the subbasin scales as shown in Figure S2, including the sources of their specific raw datasets are publicly available and can be downloaded at in the following metadata record: https://doi.org/10.6084/m9.figshare.13333796 (Strokal et al., 2021).
4 Hydrology	Natural river discharges were taken directly from the MARINA-Multi-Global-2.0 model on the sub-basin scale (at the outlets of the sub-basins) for 2010 and 2050 (Li et al., 2022), which was produced by the VIC hydrological model (van Vliet et al., 2016). Data for actual water discharge in 2010 were derived based on natural river discharge from MARINA-Multi-Global-2.0 and the ratio ($R=Q_{act}/Q_{nat}$) between actual and natural river discharges from the Global *NEWS-2* model. R >=1 are set to 1. R<=0 are set to 0.05. Actual river discharges in this study are thus derived by multiplying natural river discharge with R. For 2010, R are calculated using Global *NEWS-2* for 2000 assuming no big changes in the ratio between 2000 and 2010. For 2050, actual river discharge were derived based on the natural river discharge from MARINA-Multi-Global-2.0 and the fraction of water consumption (FQrem = 1 – Q_{act}/Q_{nat}). FQrem for SSPs in 2050 is derived based on FQrem in 2010 and changes (%) in population between 2010 and 2050 ($FQrem_{2050}$ = 0.25*percentage change in population*$FQrem_{2010}$). Here the parameter of 0.25 was used assuming water consumption does not increase/decrease equally to population change.
5 Other parameters	The model inputs to calculate the function of annual runoff are taken from MARINA-Multi-Global 2.0 model, followed an uncalibrated approach. Details for this are available in Supplementary Table S5 by Li et al. (2022. The rest of the other parameters were directly taken from the MARINA-Nutrients-China-2.0 model (Wang et al., 2020a).

[i] Direct discharges of animal manure as a point source of N pollution that is only relevant for China in 2010.

MAgPIE

The Model of Agricultural Production and its Impact on the Environment (MAgPIE) is a global land-system modelling framework (Dietrich et al., 2019) that can be used to simulate long-term scenarios for the global land and food system. It is a recursively dynamic model that simulates how food, feed and material demand can be fulfilled under different possible future pathways. Demand is driven by demographic and economic development across different regions that is provided as exogenous input to the model through the Shared Socioeconomic Pathways (SSPs) (O'Neill et al., 2017; O'Neill et al., 2014). Per-capita food intake, dietary composition (e.g., share of meat and dairy products) and food waste are driven by per-capita income (Bodirsky et al., 2020). Food intake also depends on demographic factors (population size, sex and age structure and physical activity level) (Bodirsky et al., 2020). First and second-generation bioenergy demand enters the model as exogenous input and can be varied depending on the mitigation efforts assumed (Klein et al., 2014; Kriegler et al., 2017; Popp et al., 2011). The demand for feed depends on the production of livestock products as well as region-specific feed baskets. Feed efficiency and feed basket composition are changed in a consistent manner based on exogenous assumption of improvements in milk and meat yields per animal (Weindl et al., 2017b). The production of processed commodities like oil, oilcake, sugar, molasses or ethanol also causes demand for primary crop commodities based on fixed historical conversion factors.

The model then estimates the extent and distribution of agricultural land (cropland and pastureland), forest areas and other natural land for the future until the year 2100. To determine optimal land-use patterns, MAgPIE follows a constrained cost minimization approach considering endogenous investment in agricultural technologies and intensification as well as international trade between regions (Popp et al., 2011). Depending on the assumed socio-economic pathway, trade is fully open or restricted by national self-sufficiency rates. Similarly, depending on the assumed mitigation policy, greenhouse gas emission prices that incentivize afforestation and emission reductions are determined in exogenous scenarios and affect global land use patterns. Climate impacts affect agricultural production in MAgPIE via changes in biophysical inputs like crop irrigation water requirements, water availability and crop yields. These biophysical inputs are provided by the Lund-Potsdam-Jena managed Land (LPJmL) global vegetation and hydrology model (Schaphoff et al., 2018; Von Bloh et al., 2018) using the IPSL-CM5A-LR climate model and considering CO2 fertilization. MAgPIE adapts to changes in biophysical conditions through climate change by changing management (e.g., intensification), expanding cropland areas or irrigated areas, re-allocating crop- and pasture land or adapting international trade.

Organic N inputs to cropland soils are estimated based on the method described in (Bodirsky et al., 2012; Bodirsky et al., 2014) and include manure, crop residues, atmospheric deposition, biological fixation by free-living microorganisms and change in soil organic matter. For pasture soils, they include manure, atmospheric deposition and biological fixation. Based on the required production, the amount of nitrogen harvested in crop biomass is estimated; subtracting the amount of N by biological fixation and N in seed provides the soil uptake. For croplands, we estimate the soil nitrogen uptake efficiency (SNUpE, Equation S5) and assume an exogenous trajectory for SNUpE for the future that is in line with the (Kanter et al., 2020) targets for 2030 and 2050 (see Table S3). In the policyHigh scenario, which defines only a target for 2030, we assume further improvement in SNUpE until 2050. For pastures, we estimate the nitrogen use efficiency (NUE, Equation S6). We assume that pasture NUE remains constant as in 2010 in all scenarios. Inorganic fertilizer is estimated as the necessary budget closure to fulfill plant requirements under a given SNUpE or NUE and under given quantities of organic fertilizers.

$$\text{SNUpE} = \frac{\text{soil\_nitrogen uptake}}{\text{soil\_inputs}}$$

$$= \frac{\text{crops and residues} - \text{biological fixation} - \text{seed}}{\text{organic inputs} + \text{inorganic fertilizer}} \quad (\boldsymbol{Eq\ S5})$$

$$\text{NUE} = \frac{\text{grazed\_biomass}}{\text{organic N inputs} + \text{inorganic fertilizer}} \quad (\boldsymbol{Eq\ S6})$$

Manure excreted by livestock is based on the feed baskets by Weindl et al. (2017a accounting for the reactive nitrogen (Nr) incorporated into the slaughtered animal based on the method in (Weindl et al., 2017b). The excretion calculation differentiates "grazing", "cropland-grazing", "confinement" and "collected for fuel" based on the feed mix. Excreted manure in confinement is distributed across different animal waste management systems based on regional data from the IPCC. The share of excreted nitrogen within stables of different animal waste management systems (e.g. "digester", "daily_spread", "traditional") for different livestock categories (ruminants (for milk and meat), chicken (for meat and egg), pig (for meat)) are derived from the IPCC Guidelines for National Greenhouse Gas Inventories (Eggleston et al., 2006) for each world region in the year 2010. The share of manure burned as household fuel starts of at a regionally different level (based on Eggleston et al. (2006) and converges to 0 when countries become high-income countries. Emissions from animal waste management are based on the fraction of each AWMS within manure management, with each AWMS having its own emission factors.

MAgPIE estimates nitrogen budgets on the level of 18 global world regions. In the post-processing of the model, these budgets are downscaled to 0.5° grid level. The downscaling procedure takes into account production and land-use-patterns. These land-use patterns are estimated within MAgPIE at the level of 200 simulation units that consist of grid cells with similar bio-physical conditions, and are downscaled to 0.5° based on existing cropland, suitable croplands and LPJmL yield patterns.

Land patterns are downscaled from cluster to 0.5° as follows: First, for all land types that show a reduction of area from one time step to another, we calculate a relative reduction factor for each low-resolution cluster. Second, we apply the cluster-level reduction factor to the 0.5° land patterns of the previous timestep (using historical data for the first projection timestep). Third, we calculate for all expanding land pools the share of each individual expanding land type in the sum of all expanding land types in a cluster cell. Fourth, we use this share to fill the area that was reduced in step 2. This method makes sure that the total of each land pool is identical on 0.5° and on cluster level, while no land pool exceeds the cell size (which could happen if proportional changes were applied to the previous 0.5° pattern).

After crop area was downscaled to 0.5°, we downscaled production under consideration of the relative yield patterns at 0.5° within each cluster. The relative yield patterns are derived using the crop yield simulations from the LPJmL model that assume homogeneous management intensity within all 0.5° grid cells. We multiply the downscaled crop area with the yields under homogenous management to derive a production potential. We use this potential as disaggregation weight to derive 0.5° production patterns from cluster-level production projections. These differ in sum from the production potential under homogenous management as current production stays sometimes below the production potentials, but also as the MAgPIE model simulates future yield improvements that can extend current production potentials.

Manure availability is disaggregated differently for ruminant and monogastric livestock. Manure of ruminants is split based on the nitrogen feed intake into a pasture and a cropland fraction. The pasture fraction is assumed to be excreted on pastures based on pasture production, such that higher-yielding pastures also receive more manure. The cropland fraction was disaggregated using Nr in crop production as weight (yet excluding second generation bioenergy crops which are not used as feed). Manure allocation for monogastric livestock differs by economic development. In low-income regions, we disaggregate the manure to 0.5° using the built-up area as disaggregation weight because in low-income countries monogastrics are mostly kept in extensive systems close to the human population (Gilbert et al., 2015). In high-income regions, we assumed that monogastric production takes place close to the feed production, so we used cropland as disaggregation weight. For middle-income regions, we interpolate between both approaches.

After downscaling crop area, crop production and manure availability, we recalculate the harvested nitrogen and organic inputs on 0.5° resolution using the method described in (Bodirsky et al., 2012; Bodirsky et al., 2014). For example, Nr in crop harvest is estimated using downscaled production and crop-specific nitrogen contents, and biological fixation by free-living nitrogen fixers is estimated based on downscaled crop area.

Because no (non-modeled) global subnational data on fertilizer application exists, we assume that farmers apply fertilizer proportional to their plants' requirements while considering the local availability of organic fertilizers. Inorganic fertilizer is distributed such that the maximum SNUpE is minimized across 0.5° grid cells, while allowing for a lower SNUpE in places where organic fertilizer is abundant. To do so, we first calculate the soil uptake requirements by the plants. We use inorganic fertilizer to top-up the organic fertilizer predominantly where the SNUpE (croplands) or NUE (pastures) would otherwise be highest without additional inorganic fertilizers. This methodology is used to model inorganic fertilizer distribution because organic fertilizers are unequally distributed, with their availability often exceeding plant requirements in areas of high manure and crop residue availability, and their redistribution (e.g. by the transport of manure across cells) is not simulated.

Cropping systems with leguminous crops often have a higher NUE (Lassaletta et al., 2014; Smil, 1999; Swaney et al., 2018), as biological fixation occurs within plant roots and is not subject to leaching, denitrification and volatilization before harvest. Our disaggregation approach for croplands takes account of this by using SNUpE ((Harvest-Biological Fixation-Seed) / (Organic and Inorganic Soil Inputs)) instead of NUE (Harvest / (Organic and Inorganic Soil Inputs + Biological Fixation)). With the same SNUpE, 0.5° grid cells with leguminous crops therefore have a higher NUE and receive less inorganic fertilizer than cells with without symbiotic nitrogen fixation. For pastures, we use NUE as we cannot separate symbiotic fixation within plants from the fixation by free-living organisms. Cropping systems with high manure inputs have lower requirements of inorganic fertilizer due to substitution, yet also a lower NUE (Swaney et al., 2018). Our method accounts for the substitution effect, but not for the lower fertilizer equivalence of manure. This, in combination with the rather homogenous distribution of livestock across croplands, leads to an underestimation of the heterogeneity of the Nr losses in space.

Table S3 Nitrogen use efficiency (NUE) estimated for pastures in 2010 and Soil nitrogen uptake efficiency (SNUpE) estimated for croplands in 2010, as well as assumed future SNUpE trajectories for 2050, in line with (Kanter et al., 2020). Future trajectories include SSP1, SSP2, SSP5. SSP is short for Shared-economic pathways. Details of the scenarios are available in Table S11. Pasture NUE remains constant in all scenarios.

	Pasture NUE	Cropland SNUPE			
Year		2010	2050	2050	2050
Scenario			SSP1	SSP2	SSP5
Rest of Latin America	0.71	0.51	0.75	0.70	0.51
Rest of Asia	0.80	0.40	0.70	0.60	0.40
Rest of Subsaharan Africa	0.61	0.54	0.75	0.70	0.54
Rest of Europe	0.63	0.70	0.78	0.75	0.70
Middle East and Northern Africa	0.52	0.63	0.75	0.70	0.63
Brazil, Argentina, Uruguay, Paraguay	0.76	0.58	0.75	0.70	0.58
Rest of Former Soviet Union	0.58	0.56	0.75	0.70	0.56
Australia, New Zealand	0.54	0.78	0.78	0.75	0.78
Tanzania, Uganda, Kenya, Burundi, Rwanda	0.77	0.55	0.75	0.70	0.55
Bangladesh, Nepal	0.90	0.58	0.70	0.60	0.58
South East Asia	0.69	0.52	0.70	0.60	0.52
Canada	0.56	0.74	0.78	0.75	0.74
China	0.64	0.46	0.70	0.60	0.46
Spain, France, Portugal	0.62	0.74	0.78	0.75	0.74
India	0.90	0.48	0.70	0.60	0.48
Japan, South Korea	0.79	0.66	0.70	0.60	0.66
Ukraine, Moldova	0.57	0.85	0.75	0.70	0.85
USA	0.37	0.75	0.78	0.75	0.75

Results of clean-water scarcity assessment

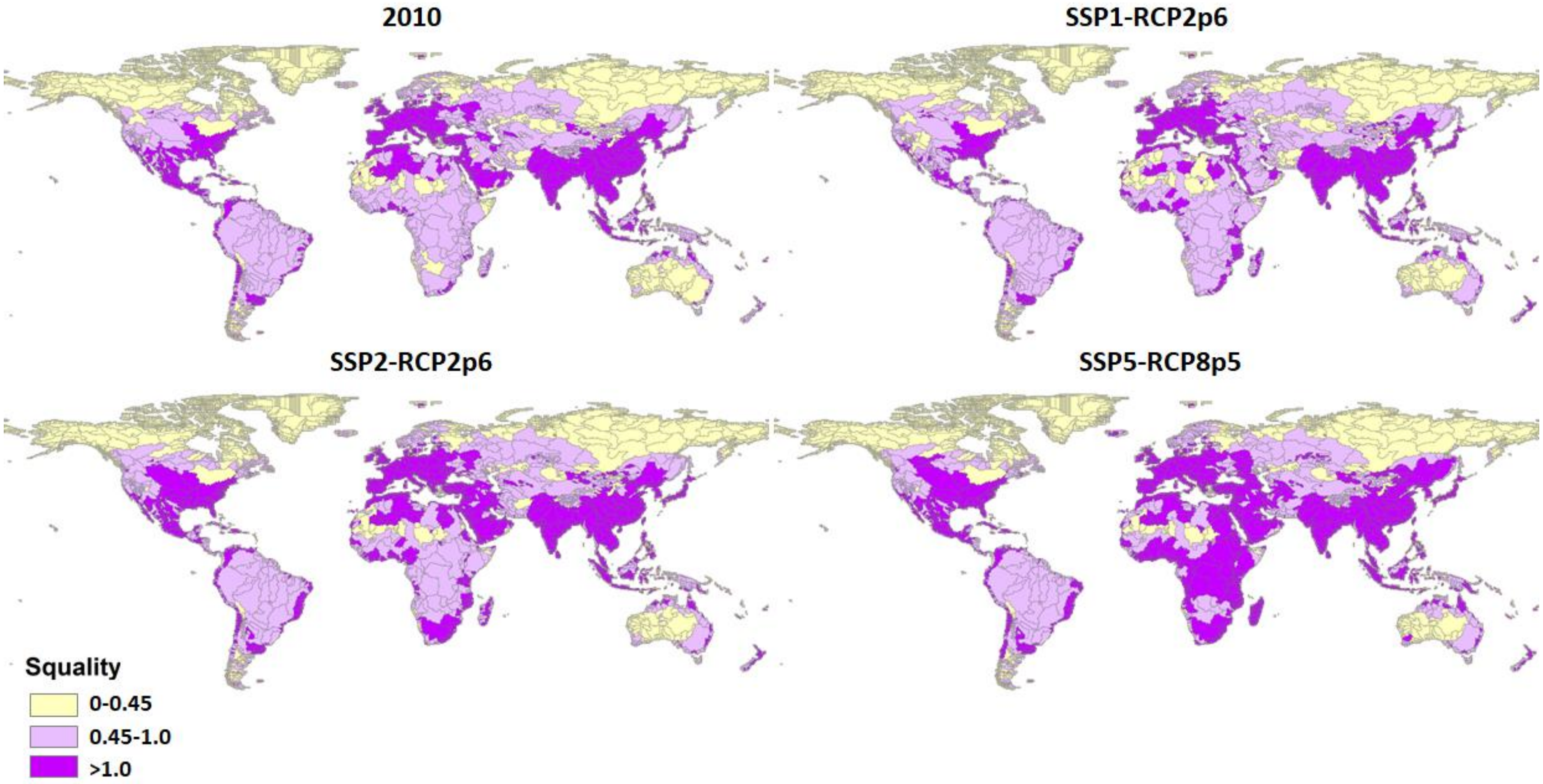

Figure S3 Quality-based water scarcity in 2010 and 2050. For 2050, water scarcity is calculated for three scenarios: SSP1-RCP2p6, SSP2-RCP2p6, SSP5-RCP8p5. Details of the scenarios based on the Shared-economic pathways (SSPs) and Representative Concentration Pathways (RCPs) are available in Tables S9-S11.

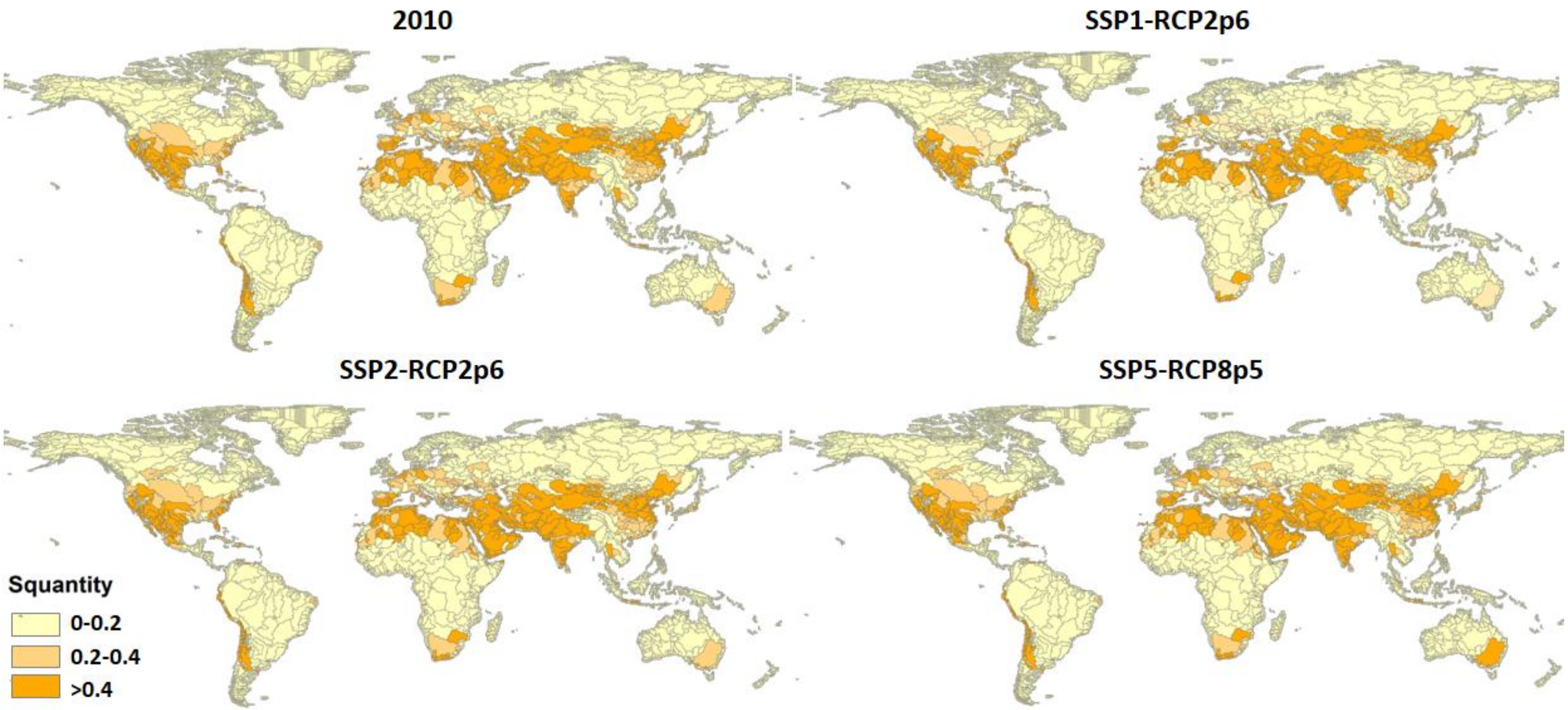

Figure S4 Quantity-based water scarcity in 2010 and 2050. For 2050, water scarcity is calculated for three scenarios: SSP1-RCP2p6, SSP2-RCP2p6, SSP5-RCP8p5. Details of the scenarios based on the Shared-economic pathways (SSPs) and Representative Concentration Pathways (RCPs) are available in Tables S9-S11.

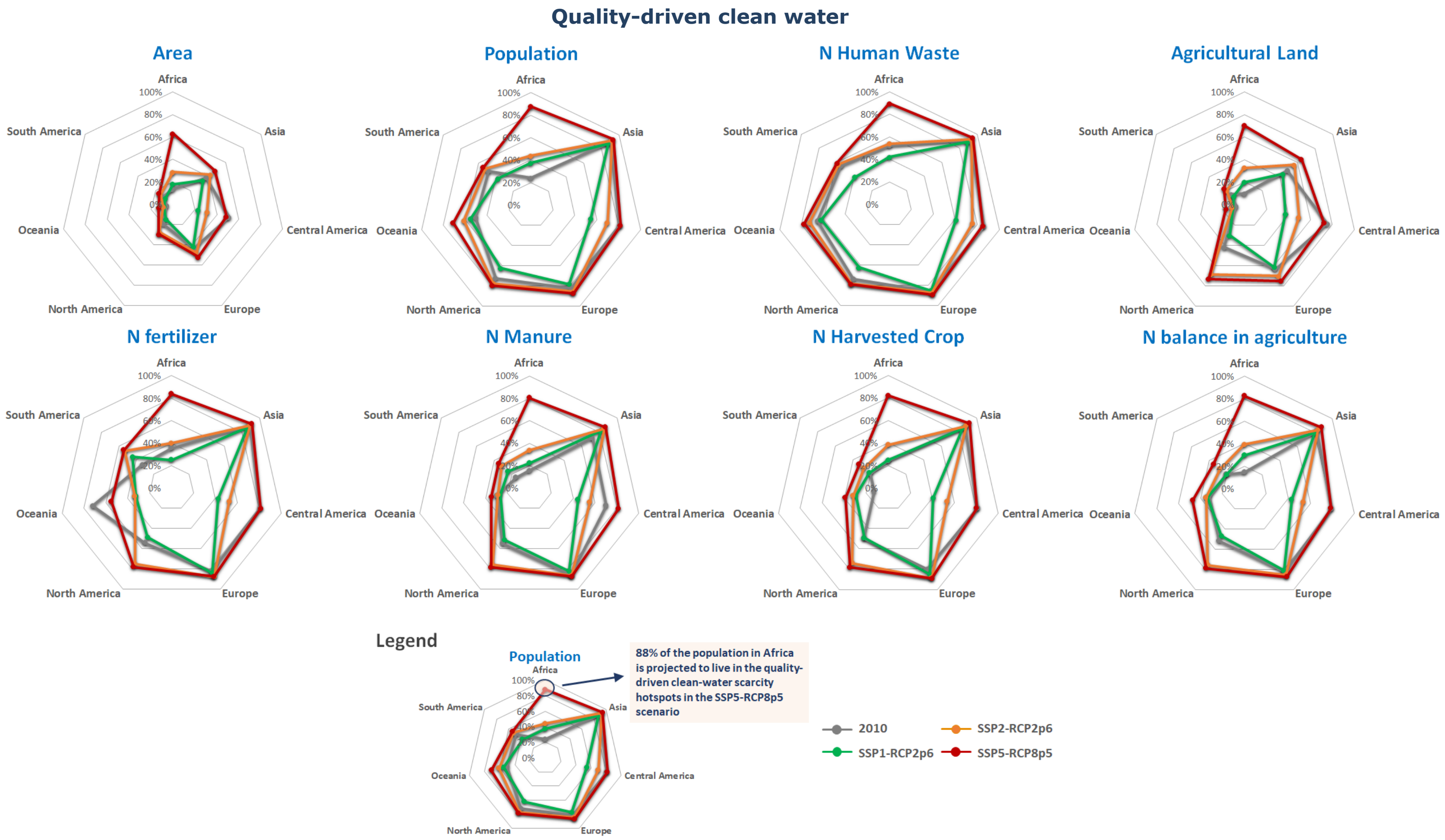

Figure S5 The shares of area (land surface), population, N (nitrogen) inputs to rivers from human waste, Agriculture land, N fertilizer application in agriculture, N manure application in agriculture, N in harvested crops, and N surplus in agriculture (defined as total N inputs to agriculture minus N outputs by crop uptake and animal grazing) in the **quality-driven** water scarcity hotspots (% of the continental total). Quality-driven water scarcity hotspots are sub-basins where the levels of scarcity for water quality are considered high in Table 2 in the main text. For 2050, water scarcity is calculated for three scenarios: SSP1-RCP2p6, SSP2-RCP2p6, SSP5-RCP8p5. Details of the scenarios based on the Shared-economic pathways (SSPs) and Representative Concentration Pathways (RCPs) are available in Tables S9-S11.

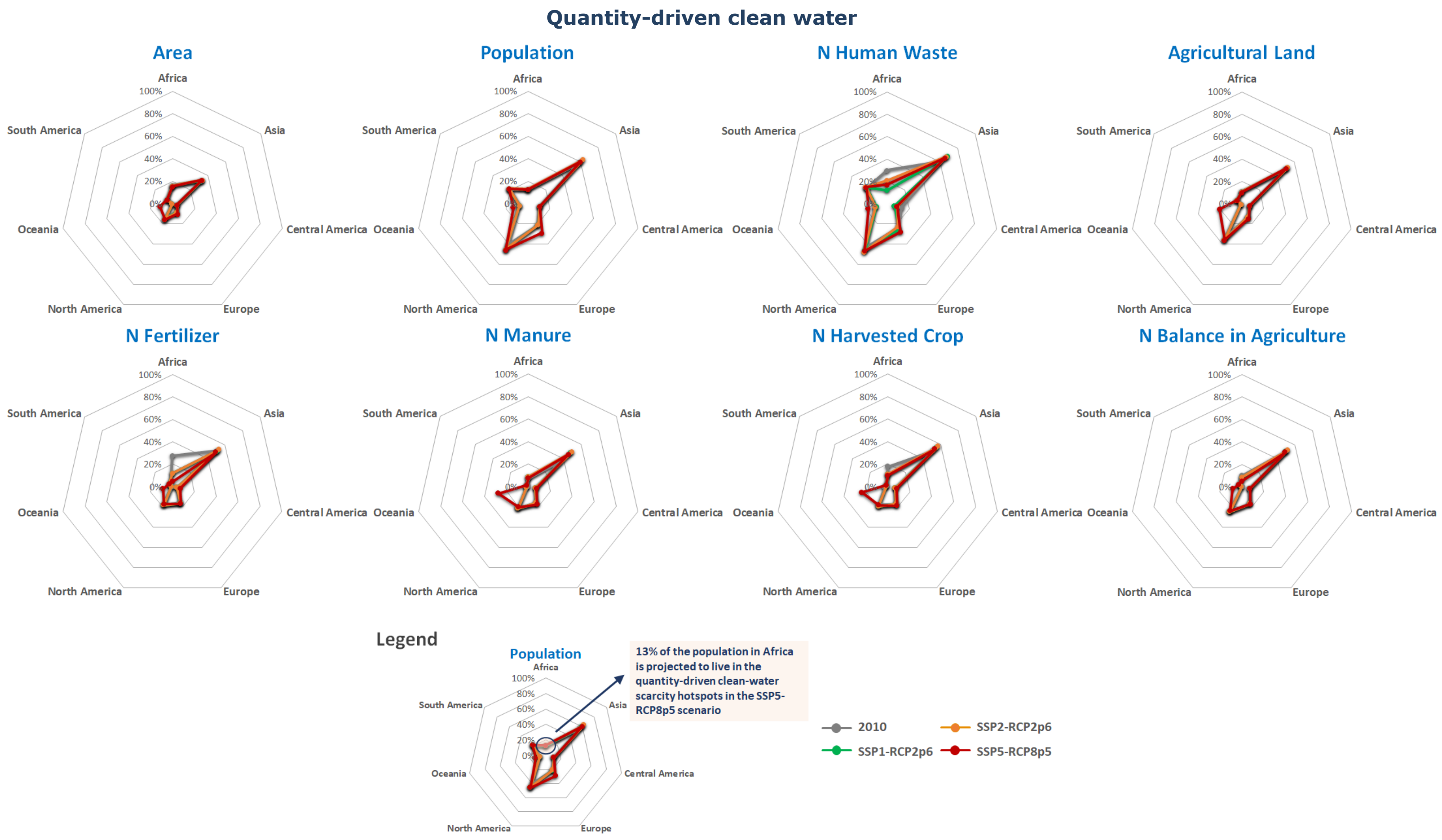

Figure S6 The shares of area (land surface), population, N (nitrogen) inputs to rivers from human waste, Agriculture land, N fertilizer application in agriculture, N manure application in agriculture, N in harvested crops, and N surplus in agriculture (defined as total N inputs to agriculture minus N outputs by crop uptake and animal grazing) in the **quantity-driven** water scarcity hotspots (% of the continental total). Quantity-driven water scarcity hotspots are sub-basins where the levels of scarcity for water quantity are considered high in Table 2 in the main text. For 2050, water scarcity is calculated for three scenarios: SSP1-RCP2p6, SSP2-RCP2p6, SSP5-RCP8p5. Details of the scenarios based on the Shared-economic pathways (SSPs) and Representative Concentration Pathways (RCPs) are available in Tables S9-S11.

Table S4 The area (land surface), population, N (nitrogen) inputs to rivers from human waste, Agriculture land, N fertilizer application in agriculture, N manure application in agriculture, N in harvested crops, and N surplus in agriculture (defined as total N inputs to agriculture minus N outputs by crop uptake and animal grazing) in the clean water scarcity hotspots. Clean water scarcity hotspots are sub-basins where either the levels of scarcity for water quantity or quality or both are considered high in Table 2 in the main text. For 2050, clean water scarcity is calculated for three scenarios: SSP1-RCP2p6, SSP2-RCP2p6, SSP5-RCP8p5. Details of the scenarios based on the Shared-economic pathways (SSPs) and Representative Concentration Pathways (RCPs) are available in the Tables S9-S11.

		Land surface	Population	N inputs to rivers by human waste	Agricultural land	N fertilizer to agriculture	N manure to agriculture	N in harvested crops	Agricultural N balance
		km^2	million people	kton	km^2	kton	kton	kton	kton
2010	Africa	6,250,249	274	283	1,805,361	1,387	2,673	1,317	2,851
	Asia	24,984,555	4,000	3,064	11,945,954	67,415	22,765	31,298	56,911
	Central America	600,418	65	85	226,532	663	570	374	836
	Europe	4,855,782	579	1,019	2,489,154	12,645	10,313	12,957	11,202
	North America	6,519,897	356	808	3,328,527	9,753	5,246	10,881	9,164
	Oceania	562,038	18	49	341,444	381	963	150	985
	South America	2,599,856	195	407	1,040,641	1,687	2,855	2,444	2,772
SSP1-RCP2P6	Africa	8,319,266	417	337	3,346,157	4,040	5,934	6,203	7,934
	Asia	25,092,254	4,003	2,542	12,797,885	81,850	40,630	65,063	71,556
	Central America	279,565	45	39	115,634	130	283	246	231
	Europe	4,688,200	549	418	2,418,392	19,197	15,398	22,448	13,584
	North America	6,589,104	337	291	3,396,482	14,320	10,672	18,009	10,949
	Oceania	788,123	19	24	476,032	892	751	994	1,515
	South America	2,273,463	155	87	964,385	3,490	3,757	4,268	2,962
SSP2-RCP2P6	Africa	9,824,962	454	778	4,329,087	5,180	7,263	7,946	9,357
	Asia	25,627,735	4,034	6,554	13,062,961	82,099	40,777	65,232	72,303
	Central America	373,294	57	135	150,574	159	348	316	284
	Europe	5,307,327	598	1,260	2,748,488	19,994	16,216	23,371	14,287
	North America	8,310,349	372	1,194	4,614,596	19,033	13,624	23,470	14,274
	Oceania	862,226	22	90	506,098	918	806	1,065	1,561
	South America	3,164,400	209	654	1,413,862	4,117	4,855	5,451	3,984
SSP5-RCP8P5	Africa	20,456,112	900	1,727	9,285,912	28,035	9,320	9,631	36,653
	Asia	27,208,418	4,060	8,156	14,203,879	123,276	31,661	63,303	108,895
	Central America	569,904	66	153	217,145	591	392	405	765
	Europe	5,735,026	607	1,544	3,086,731	21,258	16,014	23,187	17,266
	North America	8,785,377	381	1,392	4,900,281	18,516	12,862	22,723	14,789
	Oceania	2,291,668	27	133	1,632,018	2,297	1,291	1,622	3,151
	South America	3,458,629	218	688	1,602,437	6,186	4,538	5,125	6,295

Table S5 The area (land surface), population, N (nitrogen) inputs to rivers from human waste, Agriculture land, N fertilizer application in agriculture, N manure application in agriculture, N in harvested crops, and N surplus in agriculture (defined as total N inputs to agriculture minus N outputs by crop uptake and animal grazing) in the **quality-driven** water scarcity hotspots. Quality-driven water scarcity hotspots are sub-basins where the levels of scarcity for water quality are considered high in Table 2 in the main text. For 2050, water scarcity is calculated for three scenarios: SSP1-RCP2p6, SSP2-RCP2p6, SSP5-RCP8p5. Details of the scenarios based on the Shared-economic pathways (SSPs) and Representative Concentration Pathways (RCPs) are available in Tables S9-S11.

		Land surface	Population	N inputs to rivers by human waste	Agricultural land	N fertilizer to agriculture	N manure to agriculture	N in harvested crops	Agricultural N balance
		km^2	million people	kton	km^2	kton	kton	kton	kton
2010	Africa	4,185,681	246	267	1,078,418	1,219	2,386	1,130	2,237
	Asia	18,123,660	3,797	2,937	8,183,517	64,707	20,607	29,486	52,077
	Central America	597,492	65	85	225,650	661	569	373	834
	Europe	4,799,670	574	1,014	2,461,865	12,519	10,256	12,837	11,121
	North America	4,953,354	331	755	2,435,328	8,359	4,420	9,113	7,490
	Oceania	541,750	18	49	325,004	381	960	130	994
	South America	1,999,844	190	399	830,968	1,615	2,689	2,380	2,551
SSP1-RCP2P6	Africa	5,605,117	384	314	2,409,059	3,168	4,711	4,950	6,840
	Asia	16,145,014	3,671	2,332	7,688,603	75,966	36,997	59,508	64,114
	Central America	270,932	44	39	111,652	126	277	237	225
	Europe	4,631,006	544	414	2,396,066	18,926	15,249	22,229	13,461
	North America	3,679,247	285	248	1,777,351	11,925	8,812	14,917	8,289
	Oceania	767,835	19	24	459,592	869	718	960	1,503
	South America	1,577,811	148	83	721,187	3,313	3,577	4,059	2,691
SSP2-RCP2P6	Africa	8,907,113	449	772	3,963,204	5,064	7,088	7,736	9,088
	Asia	20,152,239	3,864	6,303	9,967,785	78,527	38,551	62,224	67,661
	Central America	364,578	56	134	148,032	156	343	310	280
	Europe	5,273,949	596	1,258	2,732,506	19,739	16,085	23,176	14,181
	North America	6,982,746	355	1,144	3,996,980	18,296	13,001	22,374	13,333
	Oceania	841,938	22	90	489,658	895	773	1,031	1,548
	South America	2,615,173	205	643	1,215,264	3,960	4,707	5,279	3,760
SSP5-RCP8P5	Africa	19,474,657	896	1,723	8,992,677	27,938	9,240	9,546	36,410
	Asia	22,707,322	3,932	7,935	11,706,608	118,798	30,130	61,432	103,328
	Central America	566,978	66	153	216,265	589	391	403	763
	Europe	5,716,192	607	1,544	3,075,329	21,241	15,996	23,166	17,252
	North America	7,431,761	363	1,332	4,271,715	17,910	12,320	21,867	13,874
	Oceania	1,213,801	25	123	741,742	1,995	727	1,020	2,682
	South America	2,900,035	213	676	1,394,628	6,014	4,405	4,977	6,008

Table S6 The area (land surface), population, N (nitrogen) inputs to rivers from human waste, Agriculture land, N fertilizer application in agriculture, N manure application in agriculture, N in harvested crops, and N surplus in agriculture (defined as total N inputs to agriculture minus N outputs by crop uptake and animal grazing) in the **quantity-driven** water scarcity hotspots. Quantity-driven water scarcity hotspots are sub-basins where the levels of scarcity for water quantity are considered high in Table 2 in the main text. For 2050, water scarcity is calculated for three scenarios: SSP1-RCP2p6, SSP2-RCP2p6, SSP5-RCP8p5. Details of the scenarios based on the Shared-economic pathways (SSPs) and Representative Concentration Pathways (RCPs) are available in Tables S9-S11.

		Land surface	Population	N inputs to rivers by human waste	Agricultural land	N fertilizer to agriculture	N manure to agriculture	N in harvested crops	Agricultural N balance
		km^2	million people	kton	km^2	kton	kton	kton	kton
2010	Africa	5,048,262	128	153	1,237,780	939	1,062	840	1,508
	Asia	15,489,530	2,369	1,932	8,303,804	37,142	10,097	17,184	32,409
	Central America	46,410	9	13	21,401	40	57	31	73
	Europe	949,583	145	276	495,039	2,435	2,035	2,877	2,214
	North America	3,415,951	185	436	1,875,499	2,749	1,720	3,294	3,563
	Oceania	32,720	3	8	23,525	0	5	30	(13)
	South America	1,099,892	80	169	301,232	115	287	121	370
SSP1-RCP2P6	Africa	4,889,946	130	91	1,319,628	1,465	1,807	2,186	1,902
	Asia	15,855,384	2,509	1,806	9,093,453	44,413	21,658	38,885	40,281
	Central America	46,410	9	4	21,219	11	39	38	39
	Europe	914,852	145	123	487,317	3,706	2,940	4,422	2,773
	North America	3,917,351	206	181	2,057,658	4,448	3,735	5,951	4,361
	Oceania	32,720	3	4	23,525	36	50	53	20
	South America	1,111,803	84	50	324,310	278	275	334	387
SSP2-RCP2P6	Africa	4,900,070	131	304	1,325,852	1,484	1,819	2,207	1,918
	Asia	16,040,045	2,639	4,588	9,210,231	46,180	22,447	40,219	41,826
	Central America	49,336	9	17	21,219	11	39	38	39
	Europe	919,471	144	321	488,383	3,707	2,942	4,426	2,776
	North America	3,907,193	206	691	2,055,036	4,445	3,732	5,946	4,357
	Oceania	32,720	3	13	23,525	36	50	53	20
	South America	1,123,508	84	274	326,519	278	275	334	389
SSP5-RCP8P5	Africa	4,755,046	130	334	1,301,198	1,493	872	1,164	2,168
	Asia	15,931,841	2,516	5,538	9,247,085	63,867	15,835	35,674	58,094
	Central America	46,410	9	16	21,375	51	37	40	71
	Europe	1,110,246	200	478	583,172	4,123	3,262	4,892	3,379
	North America	3,970,652	209	784	2,082,112	3,976	3,166	5,150	4,220
	Oceania	1,090,299	5	27	897,363	313	578	618	475
	South America	1,114,594	84	272	326,713	420	245	291	562

Table S7 The shares (% of the global total) of area (sub-basins drainage area), population, N (nitrogen) losses to rivers from human waste, agriculture land, N fertilizer application in agriculture, N manure application in agriculture, N in harvested crops, and N surplus in agriculture (defined as total N inputs to agriculture minus N outputs by crop uptake and animal grazing) in the quality-driven water scarcity hotspots 2010 and 2050. Quality-driven water scarcity hotspots are sub-basins where the levels of scarcity for water quality are considered high in Table 2 in the main text. For 2050, quality-driven water scarcity is calculated for three scenarios: SSP1-RCP2p6, SSP2-RCP2p6, SSP5-RCP8p5. Details of the scenarios based on the Shared-economic pathways (SSPs) and Representative Concentration Pathways (RCPs) are available in Tables S9-S11.

	% of the global total							
Year	Area	Population	N Human Waste	Agricultural land	N fertilizer	N manure	N Harvested Crop	N surplus in agriculture
2010	24	76	81	32	80	49	65	63
SSP1-RCP2p6	23	74	75	31	72	58	63	62
SSP2-RCP2p6	31	81	83	45	80	67	72	70
SSP5-RCP8p5	42	89	89	59	86	77	82	81

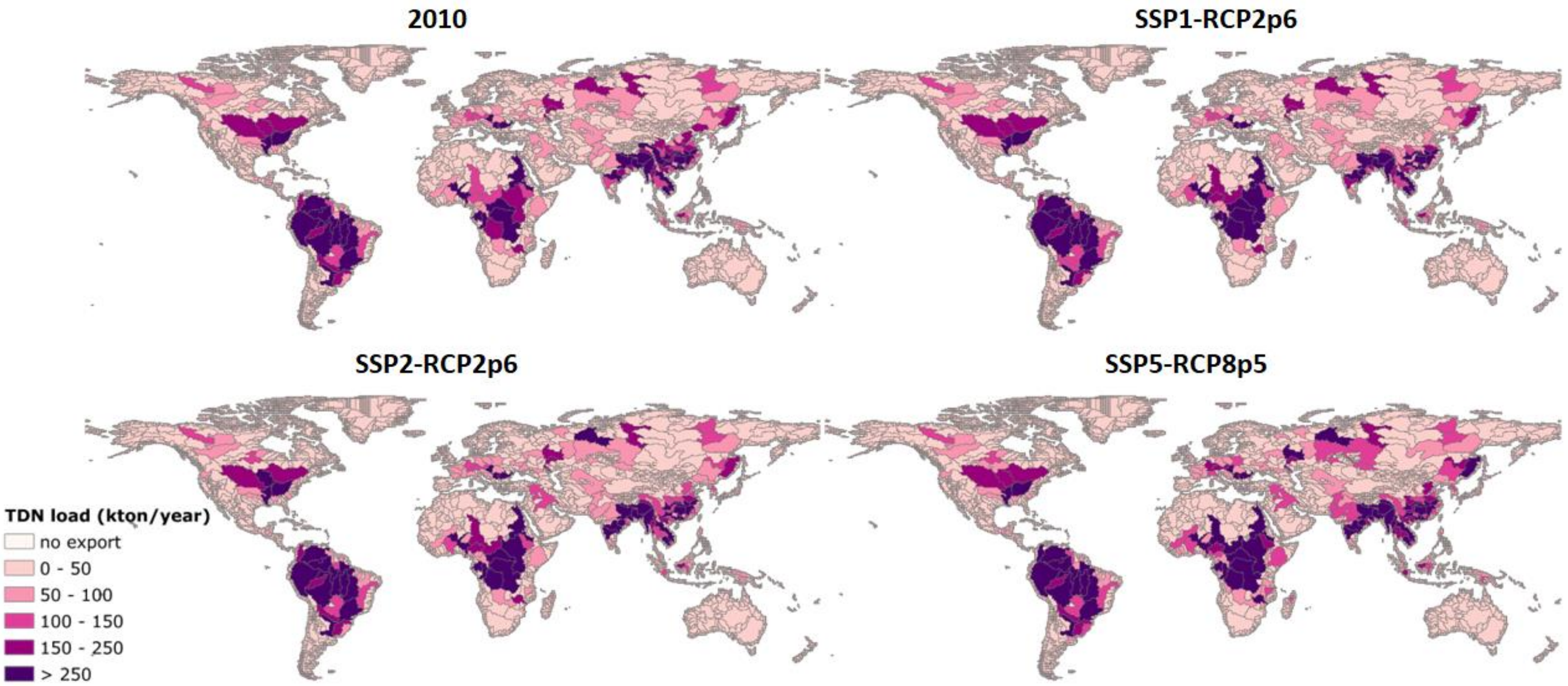

Figure S7: Total Dissolved Nitrogen (TDN) loads at the outlets of 10,226 sub-basins in 2010 and 2050 (kton/year). For 2050, TDN loads are calculated for three scenarios: SSP1-RCP2p6, SSP2-RCP2p6, SSP5-RCP8p5. Details of the scenarios based on the Shared-economic pathways (SSPs) and Representative Concentration Pathways (RCPs) are available in Tables S9-S11.

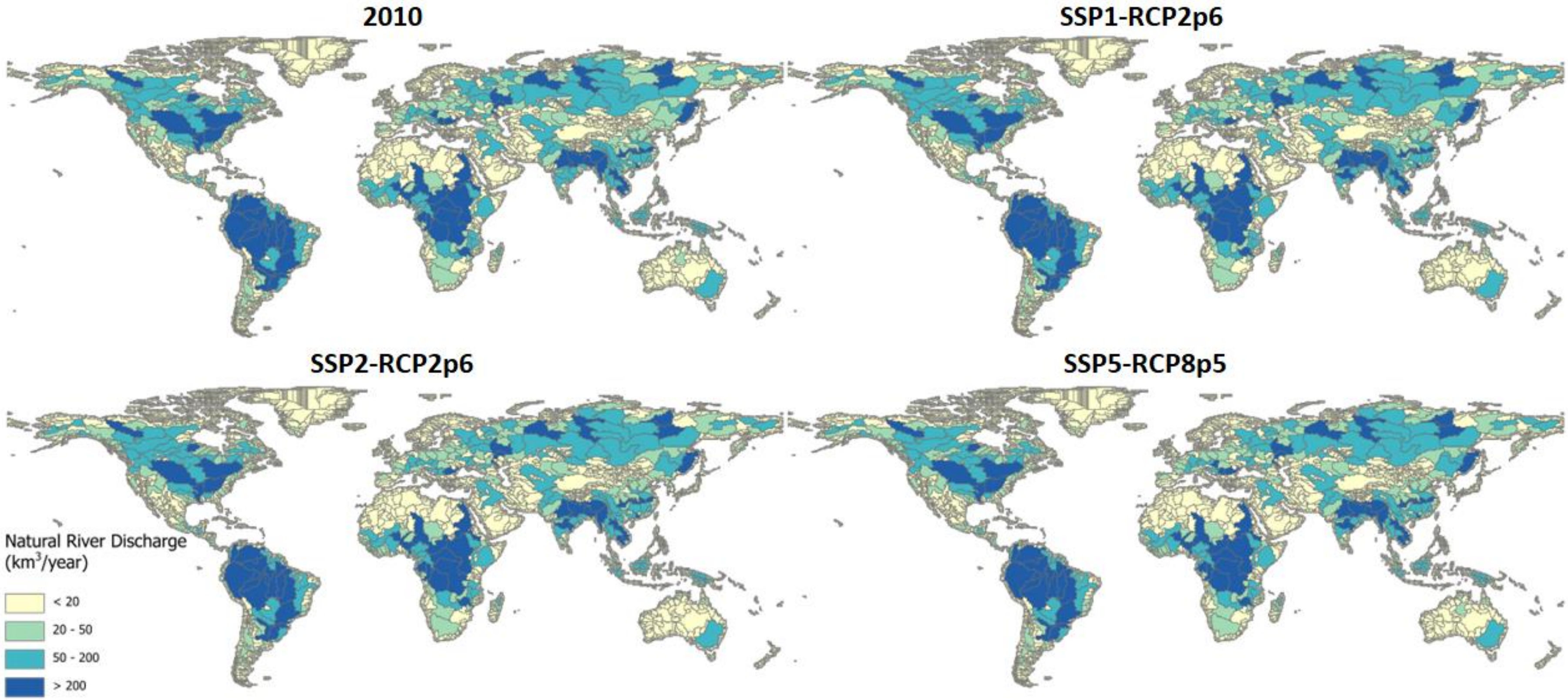

Figure S8: Natural river discharges at the outlets of 10,226 sub-basins in 2010 and 2050 (km^3/year). For 2050, river discharges are calculated for three scenarios: SSP1-RCP2p6, SSP2-RCP2p6, SSP5-RCP8p5 as explained in Table S2.

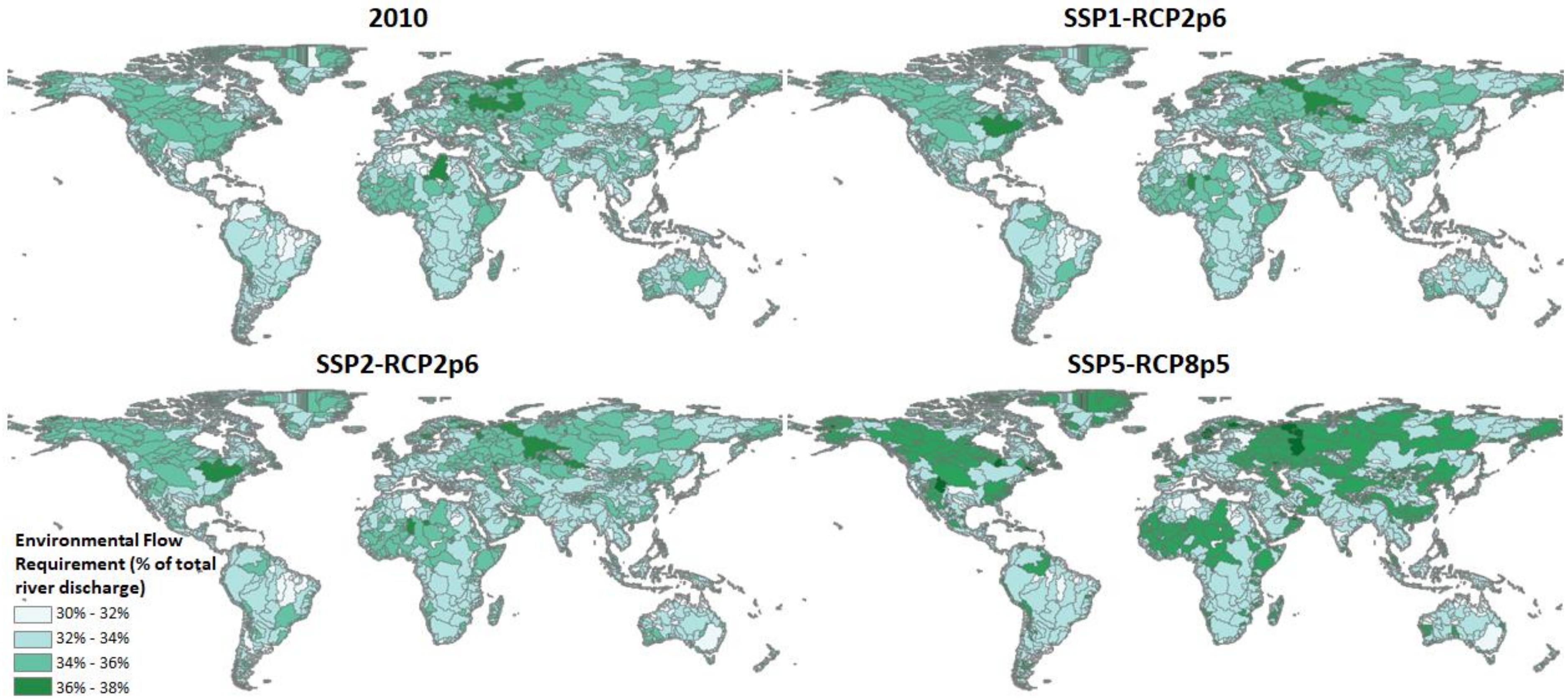

Figure S9: Environmental Flow Requirements (EFRs) for 10,226 sub-basins in 2010 and 2050 (% of total river discharge). For 2050, EFRs are calculated for three scenarios: SSP1-RCP2p, SSP2-RCP2p6, SSP5-RCP8p5 as explained in Section "Data for clean-water scarcity assessment" in this file.

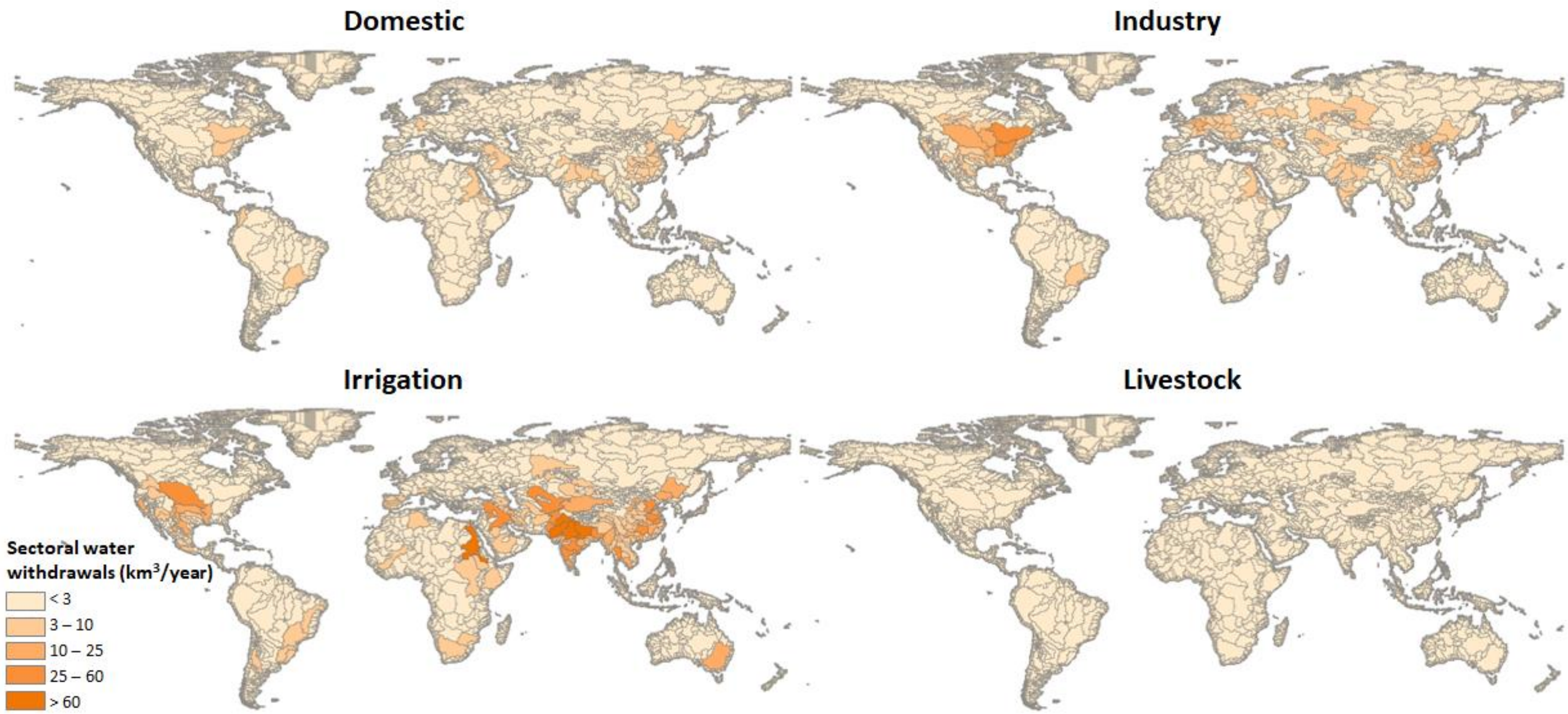

Figure S10: Sectoral water withdrawals in 10,226 sub-basins in 2010 (km^3/year).

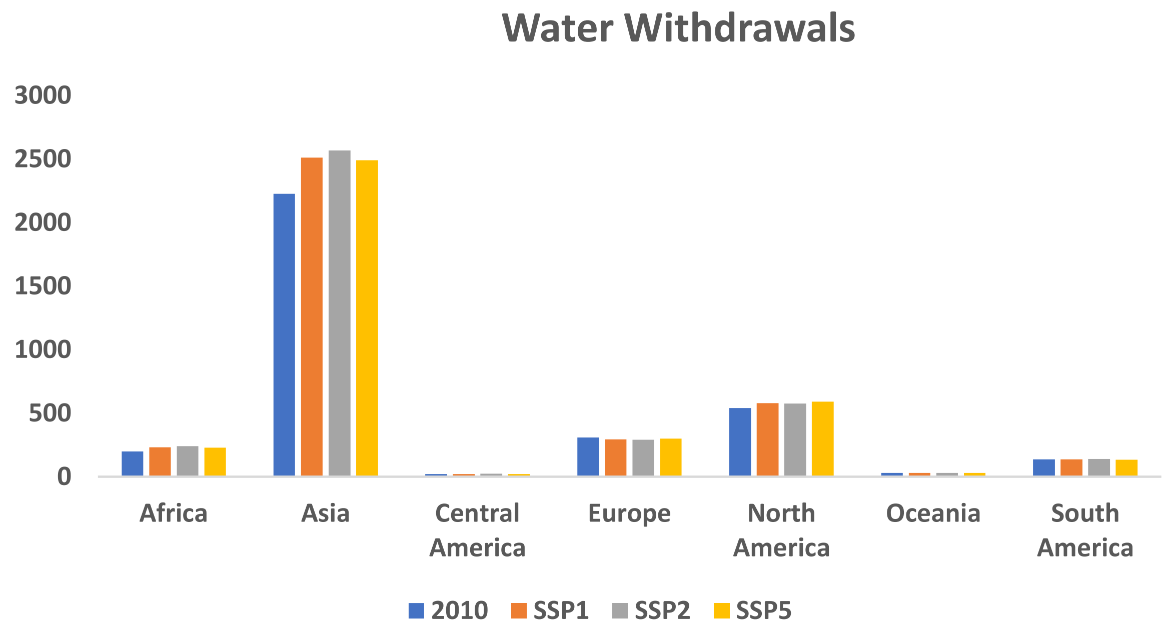

Figure S11 Total water withdrawals in seven continents in 2010 and 2050 (km3/year). For 2050, water withdrawals are derived for three scenarios: SSP1-RCP2p6, SSP2-RCP2p6, SSP5-RCP8p5. Details of the scenarios based on the Shared-economic pathways (SSPs) and Representative Concentration Pathways (RCPs) are available in Tables S9-S11.

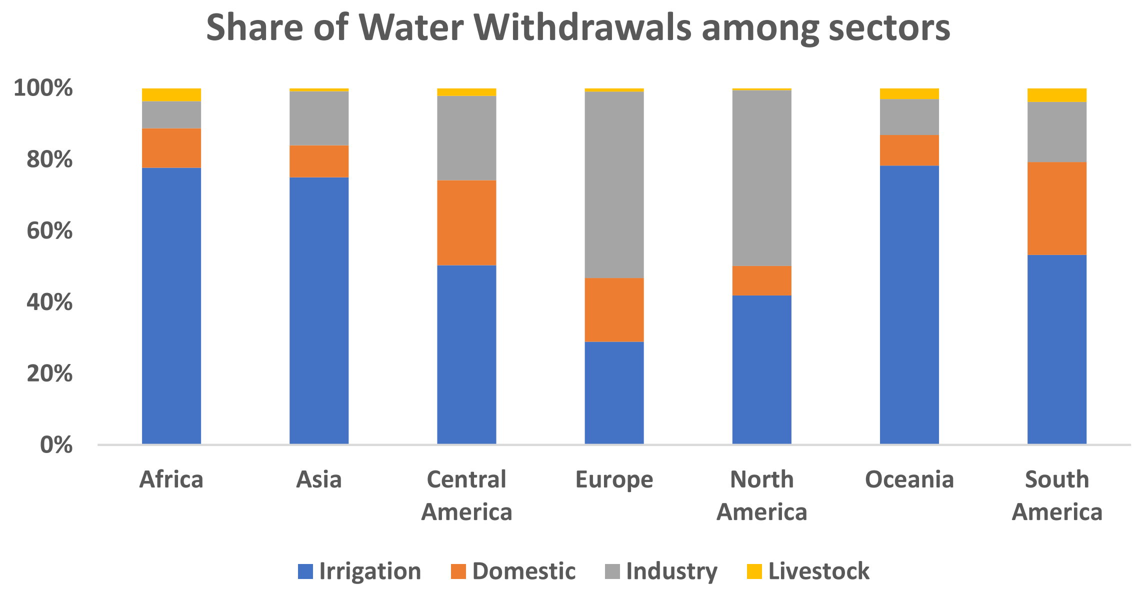

Figure S12 Share (%) of water withdrawals by sectors in seven continents in 2050.

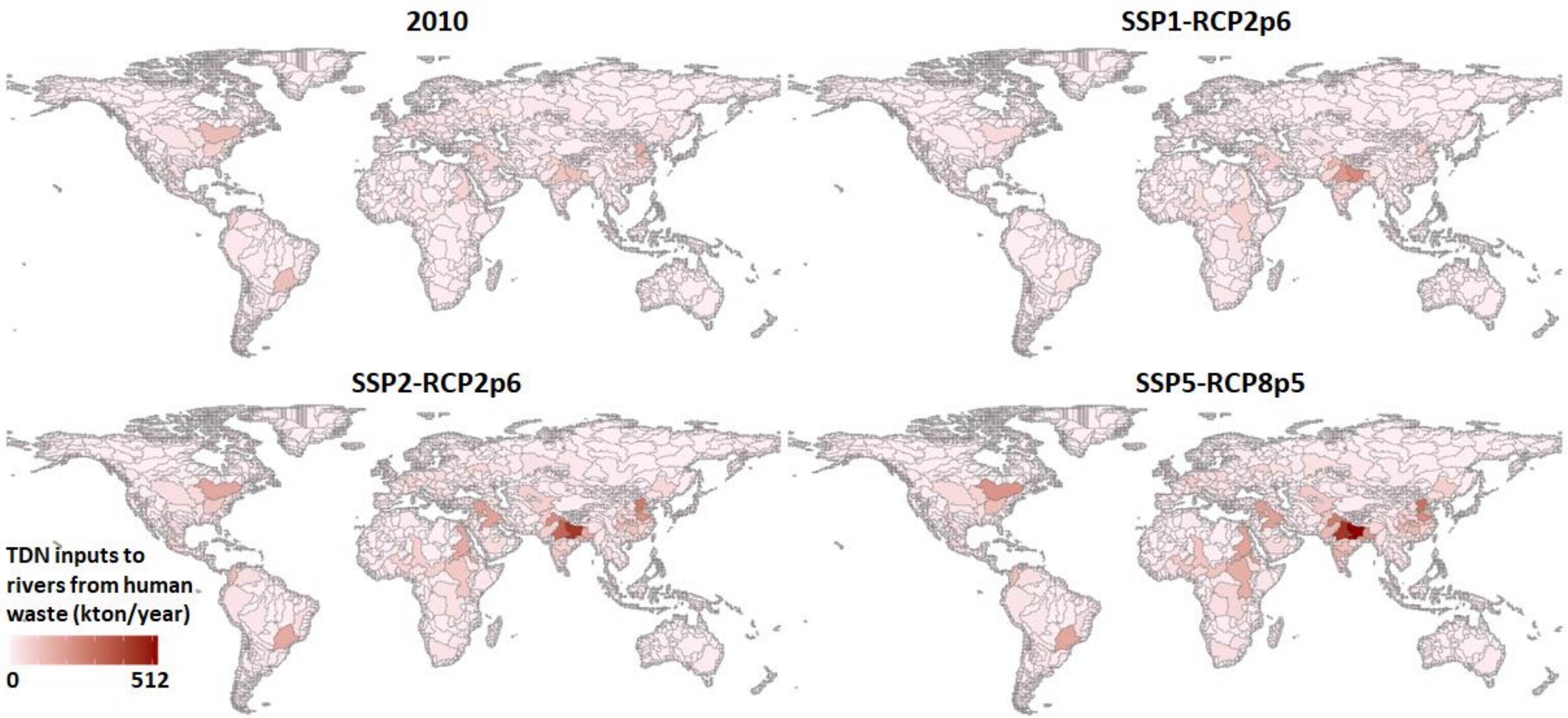

Figure S13 Total dissolved nitrogen (TDN) inputs to rivers from human waste (sum of sewage system and open defecation) for 10,226 sub-basins in 2010 and 2050 (kton/year). For 2050, data are presented for three scenarios: SSP1-RCP2p6, SSP2-RCP2p6, SSP5-RCP8p5. Details of the scenarios based on the Shared-economic pathways (SSPs) and Representative Concentration Pathways (RCPs) are available in Tables S9-S11.

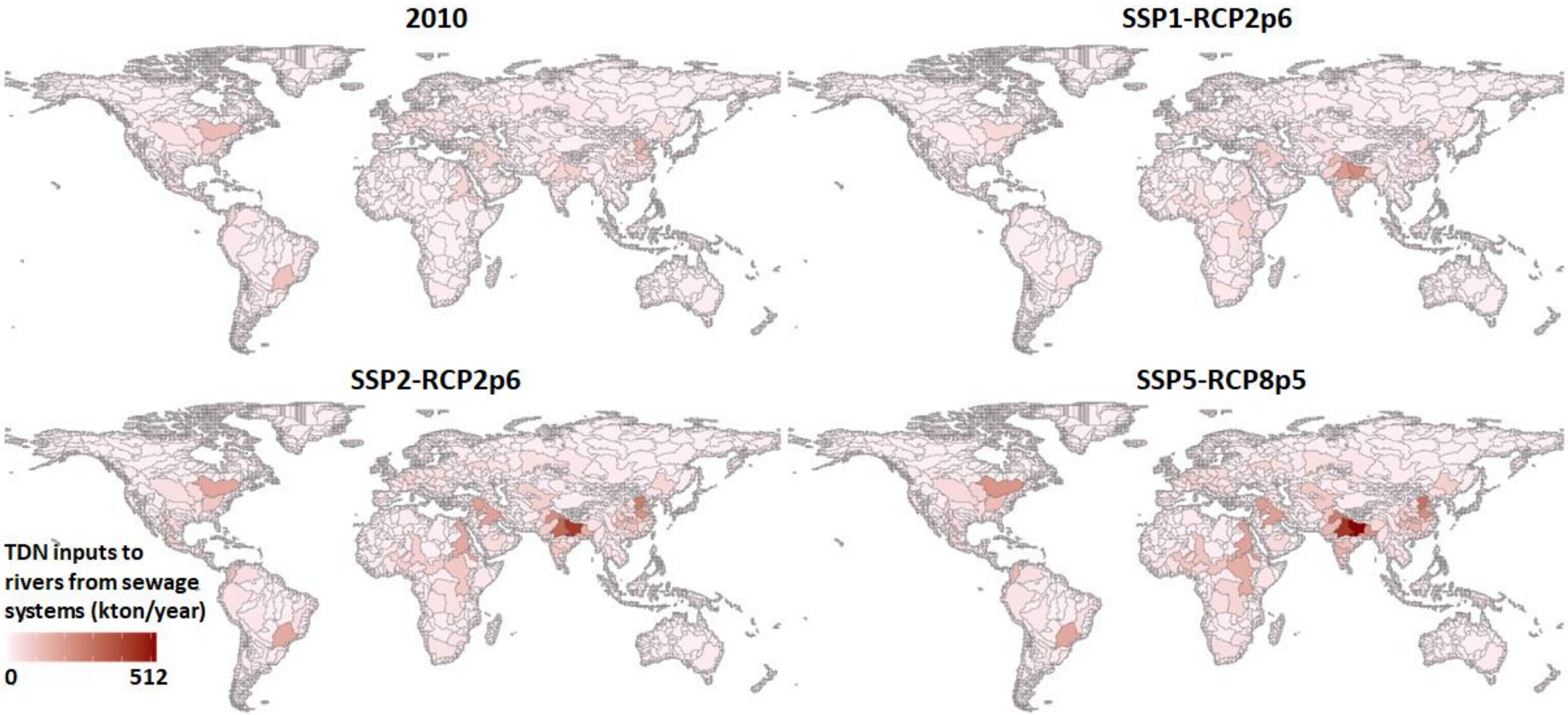

Figure S14 Total dissolved nitrogen (TDN) inputs to rivers from sewage systems for 10,226 sub-basins in 2010 and 2050 (kton/year). For 2050, data are presented for three scenarios: SSP1-RCP2p6, SSP2-RCP2p6, SSP5-RCP8p5. Details of the scenarios based on the Shared-economic pathways (SSPs) and Representative Concentration Pathways (RCPs) are available in Tables S9-S11.

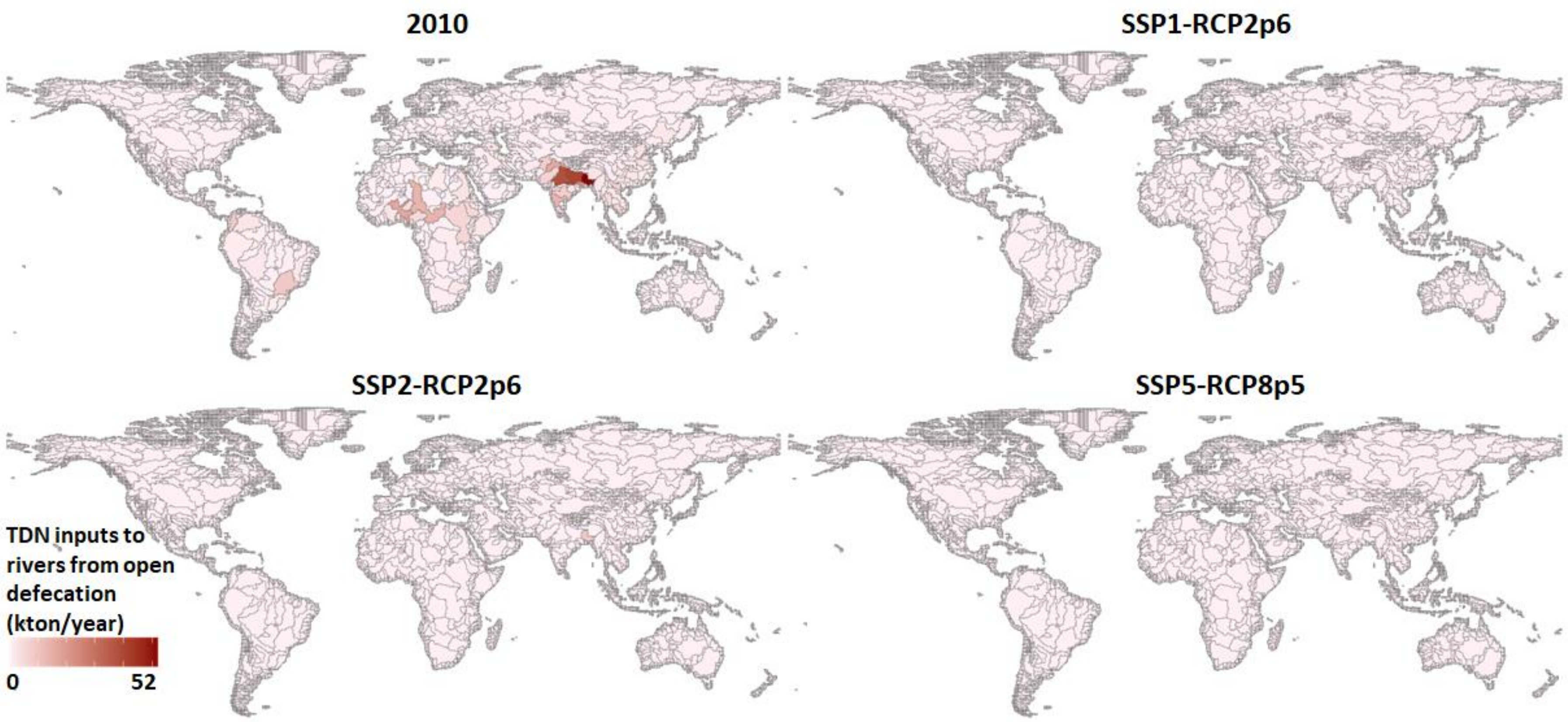

Figure S15 Total dissolved nitrogen (TDN) inputs to rivers from open defecation for 10,226 sub-basins in 2010 and 2050 (kton/year). For 2050, data are presented for three scenarios: SSP1-RCP2p6, SSP2-RCP2p6, SSP5-RCP8p5. Details of the scenarios based on the Shared-economic pathways (SSPs) and Representative Concentration Pathways (RCPs) are available in Tables S9-S11.

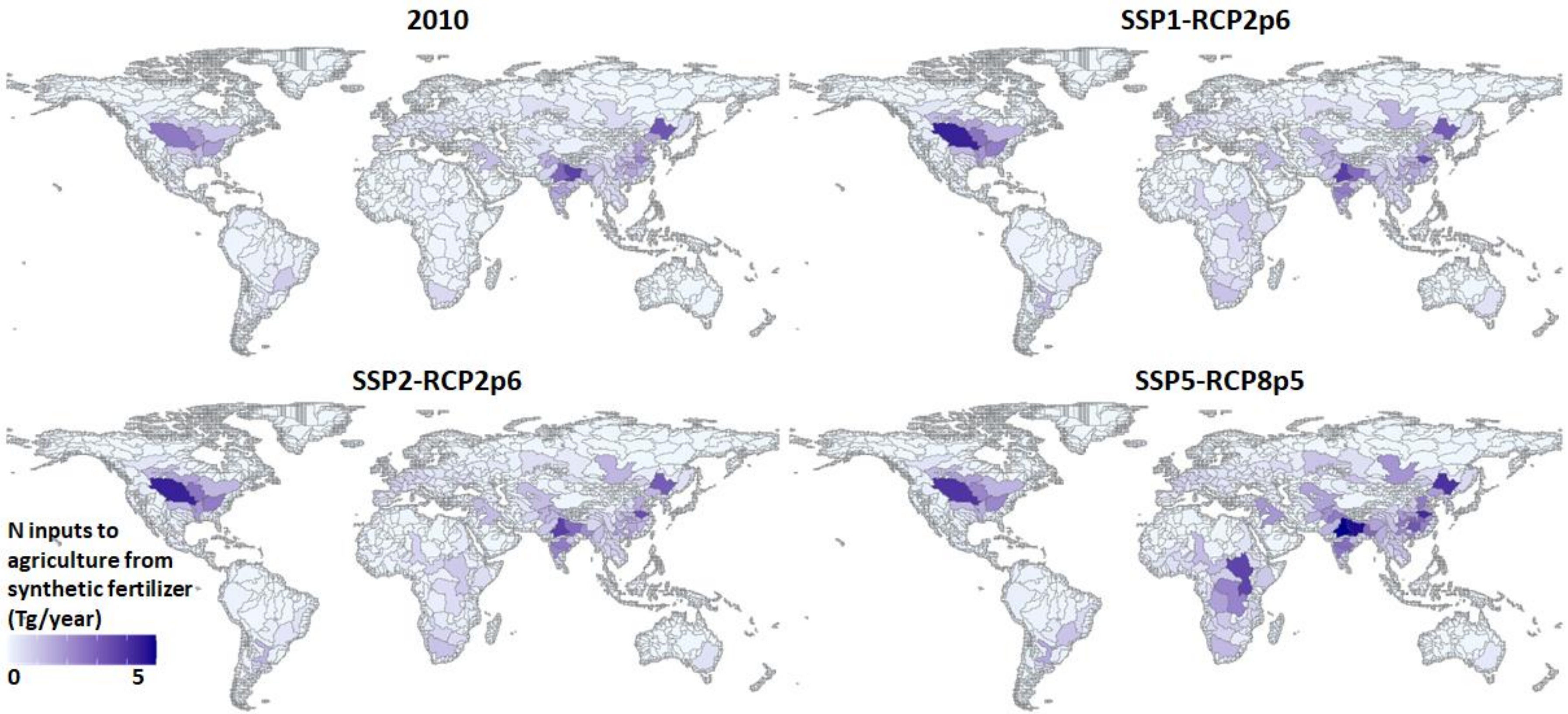

Figure S16 Nitrogen (N) inputs to agriculture from synthetic fertilizer for 10,226 sub-basins in 2010 and 2050 (Tg/year). For 2050, data are presented for three scenarios: SSP1-RCP2p6, SSP2-RCP2p6, SSP5-RCP8p5. Details of the scenarios based on the Shared-economic pathways (SSPs) and Representative Concentration Pathways (RCPs) are available in the Tables S9-S11.

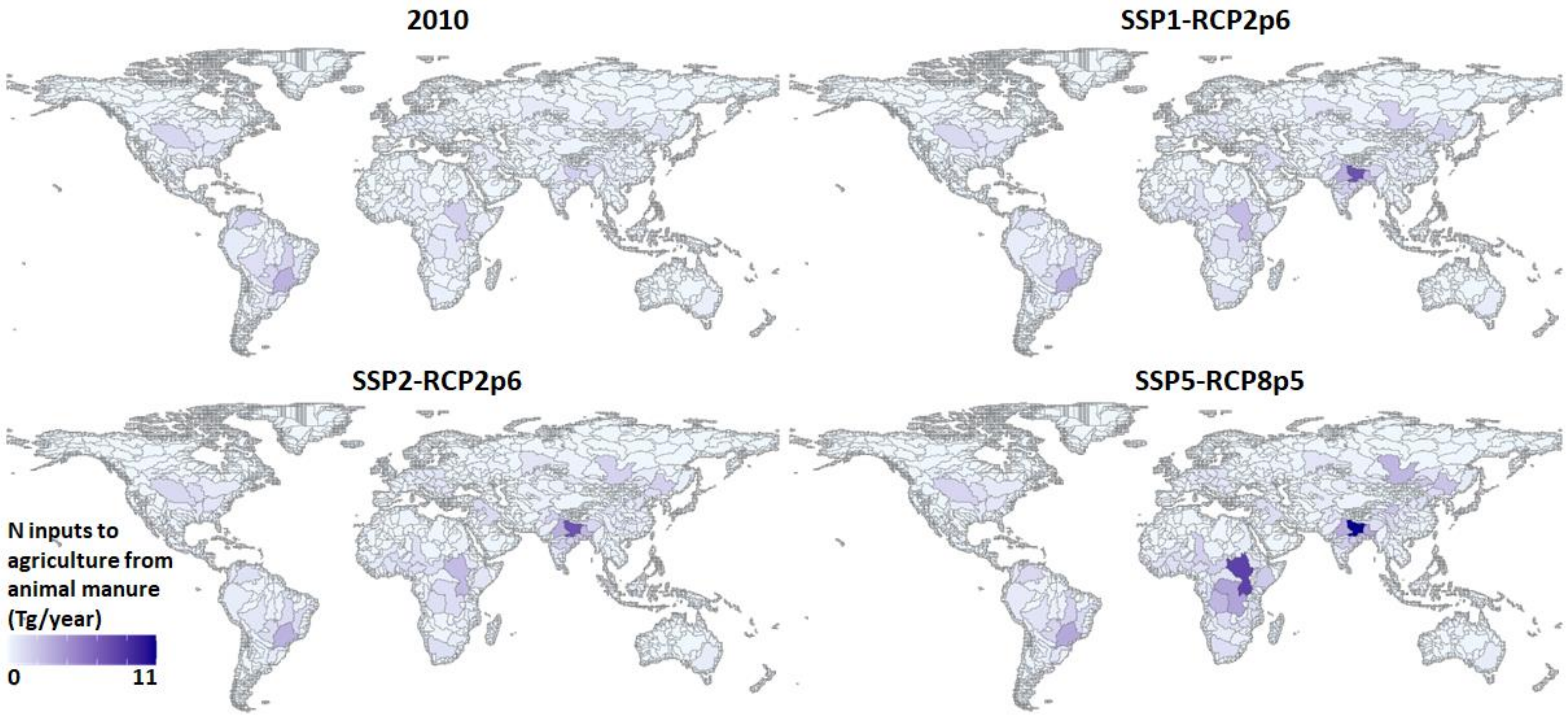

Figure S17 Nitrogen (N) inputs to agriculture from animal manure for 10,226 sub-basins in 2010 and 2050 (Tg/year). For 2050, data are presented for three scenarios: SSP1-RCP2p6, SSP2-RCP2p6, SSP5-RCP8p5. Details of the scenarios based on the Shared-economic pathways (SSPs) and Representative Concentration Pathways (RCPs) are available in Tables S9-S11.

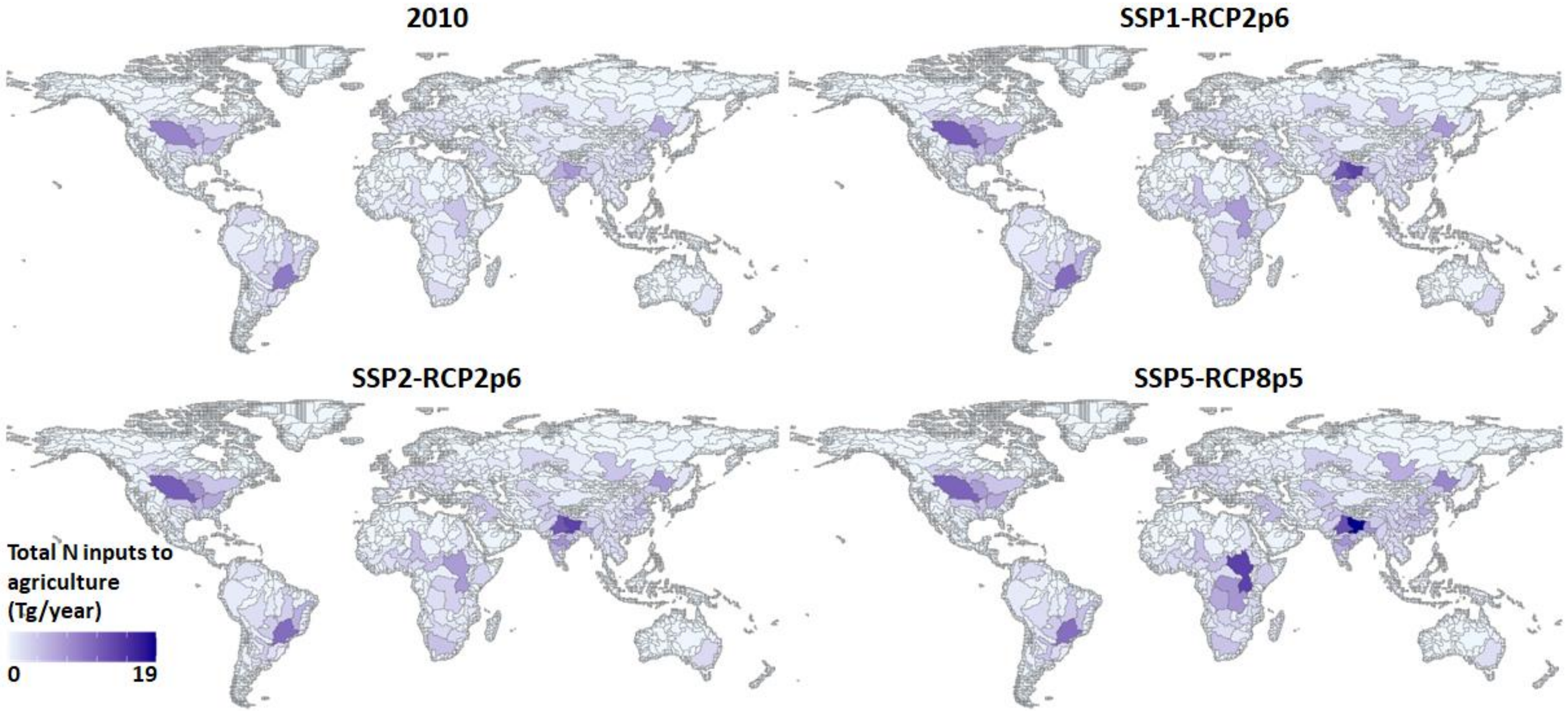

Figure S18 Total nitrogen (N) inputs to agriculture for 10,226 sub-basins in 2010 and 2050 (Tg/year). For 2050, data are presented for three scenarios: SSP1-RCP2p6, SSP2-RCP2p6, SSP5-RCP8p5. Details of the scenarios based on the Shared-economic pathways (SSPs) and Representative Concentration Pathways (RCPs) are available in Tables S9-S11.

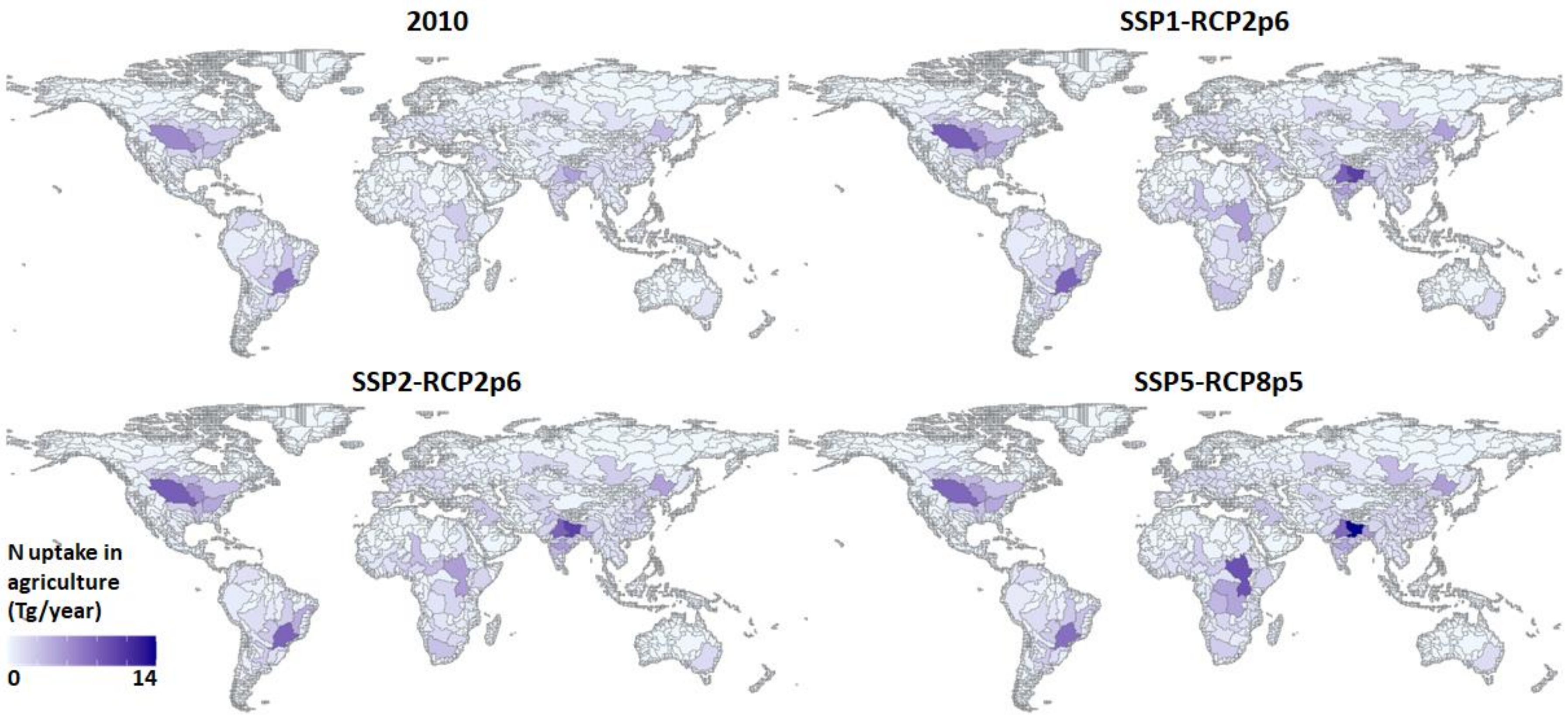

Figure S19 Nitrogen (N) uptake by crops and grasses in agriculture for 10,226 sub-basins in 2010 and 2050 (Tg/year). For 2050, data are presented for three scenarios: SSP1-RCP2p6, SSP2-RCP2p6, SSP5-RCP8p5. Details of the scenarios based on the Shared-economic pathways (SSPs) and Representative Concentration Pathways (RCPs) are available in Tables S9-S11.

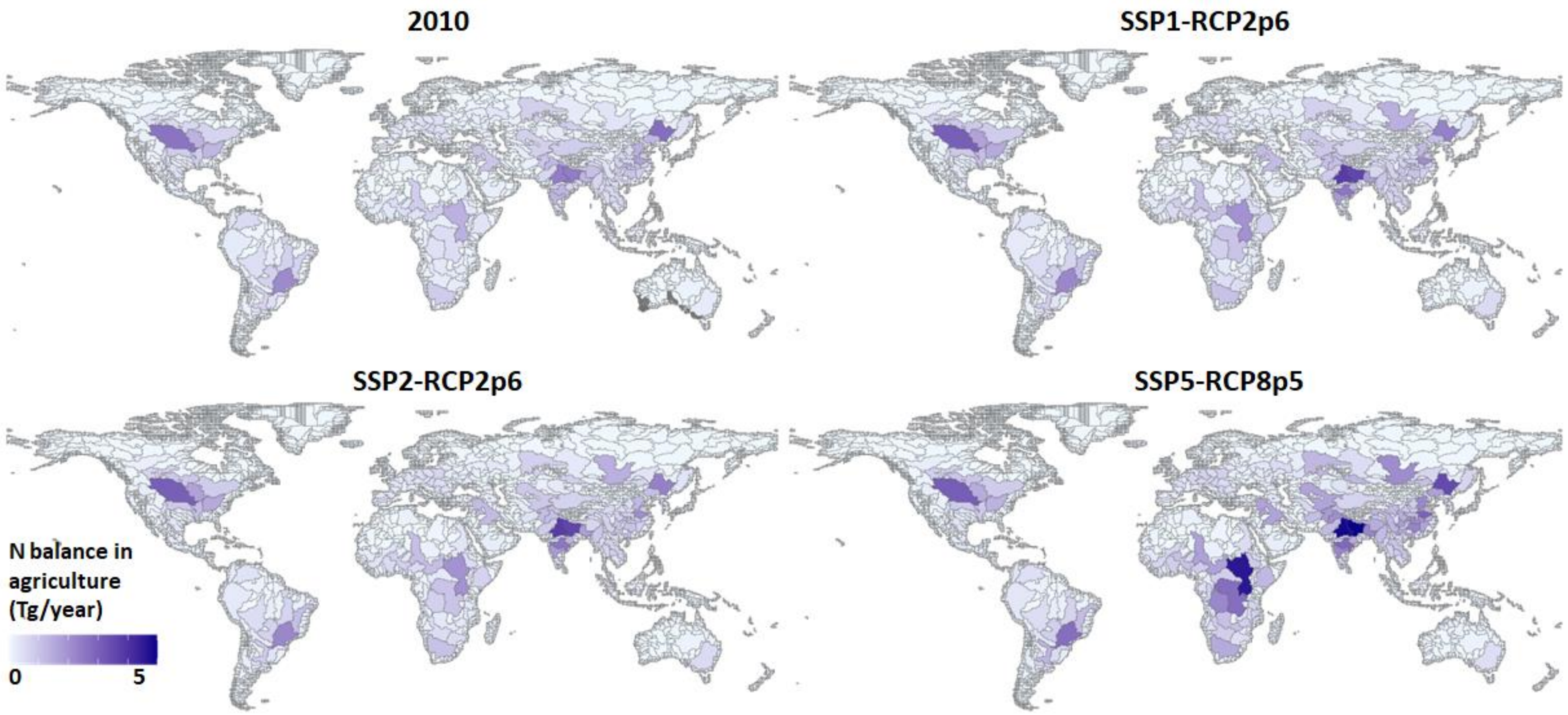

Figure S20 Nitrogen (N) balance (total inputs – uptake) in agriculture for 10,226 sub-basins in 2010 and 2050 (Tg/year). For 2050, data are presented for three scenarios: SSP1-RCP2p6, SSP2-RCP2p6, SSP5-RCP8p5. Details of the scenarios based on the Shared-economic pathways (SSPs) and Representative Concentration Pathways (RCPs) are available in Tables S9-S11.

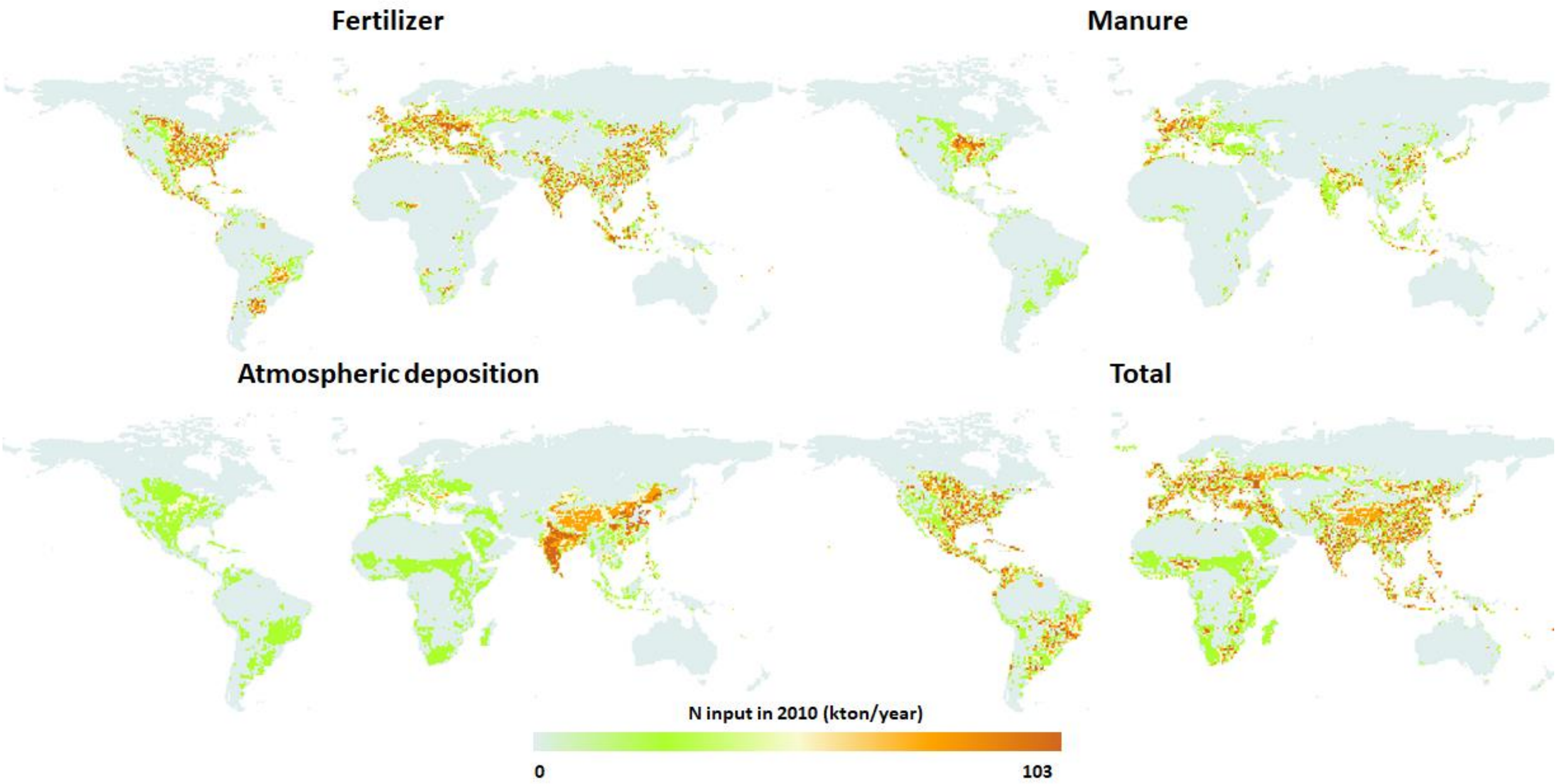

Figure S21 Nitrogen (N) inputs in agriculture simulated in MAgPIE (Model of Agricultural Production and its Impacts on the Environment) including fertilizer application in cropland and pasture, manure application in cropland and pasture, atmospheric deposition on cropland and pasture, and total as sum of fertilizer, manure and deposition in cropland and pasture.

Table S8 Nitrogen inputs to the terrestrial biosphere in Tian et al. (2022 and this study based on MAgPIE (Model of Agricultural Production and its Impacts on the Environment). The Nitrogen inputs are: fertilizer applied to cropland and pasture, manure applied/deposition on cropland and pasture, atmospheric deposition. Total is the sum of fertilizer, manure and deposition in this table.

Tian et al. (2022					This study (MAgPIE)				
Decade	Fertilizer	Manure[i]	Deposition[ii]	Total	Year	Fertilizer	Manure[i]	Deposition[ii]	Total
1990s	77	87	62	226	1995	86	78	72	236
2000s	88	92	62	242	2000	89	81	72	242
2010s	105	98	64	267	2010	112	90	84	287

[i] For the study of Tian et al. (2022, this includes manure applied on cropland and manure deposition on pasture. For MAgPI, this include manure applied on cropland, manure from stubble grazing on cropland, and manure from grazing on pasture.
[ii] The atmospheric deposition includes deposition on both agricultural and non-agricultural land

Table S9 Basis for the scenarios in 2050 in this study. SSPs stands for the Shared Socio-economic Pathways (O'Neill et al., 2017). RCPs stands for the Representative Concentration Pathways (Van Vuuren et al., 2011).

Scenario name	Land use and agriculture [i]	Urbanization and wastewater treatment [ii]	Water withdrawal [iii]	Climate impacts on hydrology [iii]
**SSP1-RCP2p6**	SSP1 [a]	SSP1 [b]	SSP1	RCP2p6 [c]
**SSP2-RCP2p6**	SSP2 [a]	SSP2 [b]	SSP2	RCP2p6 [c]
**SSP5-RCP8p5**	SSP5 [a]	SSP5 [b]	SSP5	RCP8p5 [c]

[i] detailed assumption available in Table S11 in this file;

[ii] detailed assumption available in Table S10 in this file;

[iii] detailed assumption available in section 'Data for clean-water scarcity assessment' in this file;

[a] (Kanter et al., 2020)

[b] (Strokal et al., 2021);

[c] (van Vliet et al., 2016).

Table S10 Assumptions for urbanizationa and wastewater treatment that are used in scenarios of this study. SSPs stands for the Shared Socio-economic Pathways (O'Neill et al., 2017).

Scenario name	Urbanization and	Wastewater treatment
**SSP1** [a]	**High** urbanization	**High** wastewater treatment rates
**SSP2** [a]	**Moderate** urbanization	**Moderate** wastewater treatment rates
**SSP5** [a]	**High** urbanization	**Moderate** wastewater treatment rates

[a] (Strokal et al., 2021).

Table S11 Assumptions for the nitrogen futures in landuse and agriculture that were used in the scenarios (Table S9) in this study. These assumptions were implemented in MAgPIE to produce N inputs on land used in this study. SSPs stands for the Shared Socio-economic Pathways (O'Neill et al., 2017). RCPs stands for the Representative Concentration Pathways (Van Vuuren et al., 2011).

**SSPs** (based on the SSP-RCP-N scenario (Zhang et al., 2015))	**Population** (Kc and Lutz, 2014) **& Economic** (Dellink et al., 2017) **Development**	**Trade**	**Land-use regulation**	**Livestock sector**[i]	**Diet** (Bodirsky et al., 2020; Bodirsky et al., 2015; Willett et al., 2019)	**Food waste** (Bodirsky et al., 2020; Bodirsky et al., 2015)	**N Policy (Crop)**	**N Policy (Livestock)**	**Climate Impacts**	**Climate Mitigation**
**SSP5** (SSP5-RCP8p5-PolicyLow)	**Fossil-fuel driven development**: population and GDP trajectories follow SSP5 scenario	SSP5 trends: **strong globalization** with high level of international trade	**Water protection** policy: environmental flow protection scenario activated.	**Medium** livestock **productivity**: Feed share and livestock productivity follow SSP2 GDP projections	**Meat & dairy-rich** (driven by SSP5 GDP growth and population scenarios)	**High food waste** and **overconsumption** (driven by SSP5 GDP growth scenario)	**Low ambition** N policy on cropland: Soil nitrogen uptake efficiency (SNUPE) of cropland is held constant at 2010-level (Bodirsky et al., 2014; Kanter et al., 2020) [i]	**Low ambition** N policy in livestock sector: Feed baskets follow the SSP2 scenario (moderate intensification and an aligned shift from pasture to concentrate feed). The share of each system in animal waste management follow the SSP2 storyline (Bodirsky et al., 2014) [ii].	**High impacts** (RCP8p5)	**No climate mitigation** (second generation bioenergy demand and pollutant prices follow SSP5 pathway (Kriegler et al., 2017; Van Vuuren et al., 2017))
**SSP2** (SSP2-RCP4p5-PolicyMed)	**Historical development trends**: population and GDP trajectories given by the SSP2 scenario	SSP2 trends: **medium globalization** with medium level of international trade	**No water protection** policy: environmental flow protection scenario deactivated	**Higher** livestock **productivity**: Feed share and livestock productivity follow SSP1 scenario GDP projections	**Medium** meat & dairy consumption (driven by SSP2 GDP growth and population scenarios). Food demand saturates for high incomes	**Medium food waste** and **overconsumption** (driven by SSP2 GDP growth scenario)	**Moderate ambition** N policy on cropland: Regional SNUPE of cropland reaches regional levels of Zhang et al. (2015) (table 1) by 2050, and 0.8 globally by 2100.	**Moderate ambition** N policy for livestock sector: Feed baskets follow SSP1 scenario (higher feeding efficiency and concentrates share, higher intensification, especially in low income countries)(Weindl et al., 2017). The share of each system in animal waste management follow the SSP1 storyline (Bodirsky et al., 2014) [iii]	**Moderate impacts** (RCP4p5)	**Moderate climate mitigation** (second generation bioenergy demand and pollutant prices follow SSP2 pathway (Fricko et al., 2017))
**SSP1** (SSP1-RCP4p5-PolicyHighDiet)	**Sustainable development**: population and GDP trajectories given by the SSP1 scenario	SSP1 trends: **medium globalization** with globalized institutions, but also focus on local communities	**Water protection** policy: environmental flow protection scenario activated.	**Higher** livestock **productivity**: Feed share and livestock productivity follow SSP1 scenario GDP projections	**Ambitious diet shift**: Food demand follows an exogenous scenario of a "healthy, flexitarian diets" in line with the EAT Lancet diet (Bodirsky et al., 2015; Willett et al., 2019). Additionally: **Low** meat & dairy (driven by SSP1 GDP growth and population scenarios). Food demand decreases for high incomes.	**Ambitious reduction** of food **waste**: food waste is reduced to 20% (this corresponds to half of current food waste of high income countries) by 2050. **Overconsumption** of food driven by SSP1 GDP growth scenario.	**High ambition** N policy on cropland: Regional SNUPE of cropland reaches regional levels of Zhang et al. (2015) (table 1) by 2030, and 0.8 globally by 2070.	**High ambition** N policy for livestock sector: Feed baskets follow SSP1 scenario (higher feeding efficiency and concentrates share, higher intensification, especially in low income countries)(Weindl et al., 2017). The share of each system in animal waste management follow the Good-practice scenario (Bodirsky et al., 2014) [iv].	**Moderate impacts** (RCP4p5)	**Ambitious climate mitigation** (second generation bioenergy demand and pollutant prices follow SSP1 pathway (Van Vuuren et al., 2017))

[i] In all scenarios, regional soil nitrogen uptake efficiency of pasture land is held constant at 2010-level.

[ii] AWM share: "Digester" AWMS make up a share of 0.3 in 2050 and 0.6 in 2100. The "Daily spread" AWMS share falls from 0.2 in 2020 to 0 in 2050 and stays at this level until 2100. The share of "Traditional" AWMS falls from 0.76 in 2020 to 0.7 in 2050 and 0.4 in 2100.

[iii] AWM share: "Digester" AWMS make up a share of 0.5 in 2050 and 0.7 in 2100. There is no "Daily spread" AWMS throughout the entire simulation period. The share of "Traditional" AWMS falls from 0.85 in 2015 to 0.5 in 2050 and 0.3 in 2100.

[iv] AWM share: "Digester" AWMS make up a share of 0.5 in 2050 and 0.7 in 2100. There is no "Daily spread" AWMS throughout the entire simulation period. The share of "Traditional" AWMS falls from 0.85 in 2015 to 0.5 in 2050 and 0.3 in 2100.

References

1. Bodirsky B, Popp A, Weindl I, Dietrich J, Rolinski S, Scheiffele L, et al. Current state and future scenarios of the global agricultural nitrogen cycle. Biogeosciences Discussions 2012; 9: 2755.
2. Bodirsky BL, Dietrich JP, Martinelli E, Stenstad A, Pradhan P, Gabrysch S, et al. The ongoing nutrition transition thwarts long-term targets for food security, public health and environmental protection. Scientific reports 2020; 10: 1-14.
3. Bodirsky BL, Popp A, Lotze-Campen H, Dietrich JP, Rolinski S, Weindl I, et al. Reactive nitrogen requirements to feed the world in 2050 and potential to mitigate nitrogen pollution. Nature communications 2014; 5: 1-7.
4. Bodirsky BL, Rolinski S, Biewald A, Weindl I, Popp A, Lotze-Campen H. Global food demand scenarios for the 21 st century. PloS one 2015; 10: e0139201.
5. De Vries W, Kros J, Kroeze C, Seitzinger SP. Assessing planetary and regional nitrogen boundaries related to food security and adverse environmental impacts. Current Opinion in Environmental Sustainability 2013; 5: 392-402.
6. Dellink R, Chateau J, Lanzi E, Magné B. Long-term economic growth projections in the Shared Socioeconomic Pathways. Global Environmental Change 2017; 42: 200-214.
7. Dietrich JP, Bodirsky BL, Humpenöder F, Weindl I, Stevanović M, Karstens K, et al. MAgPIE 4 – a modular open-source framework for modeling global land systems. Geosci. Model Dev. 2019; 12: 1299-1317.
8. Droppers B, Franssen WH, Van Vliet MT, Nijssen B, Ludwig F. Simulating human impacts on global water resources using VIC-5. Geoscientific Model Development 2020; 13: 5029-5052.
9. Eggleston H, Buendia L, Miwa K, Ngara T, Tanabe K. 2006 IPCC guidelines for national greenhouse gas inventories. 2006.
10. Fricko O, Havlik P, Rogelj J, Klimont Z, Gusti M, Johnson N, et al. The marker quantification of the Shared Socioeconomic Pathway 2: A middle-of-the-road scenario for the 21st century. Global Environmental Change 2017; 42: 251-267.
11. Gilbert M, Conchedda G, Van Boeckel TP, Cinardi G, Linard C, Nicolas G, et al. Income Disparities and the Global Distribution of Intensively Farmed Chicken and Pigs. PLOS ONE 2015; 10: e0133381.
12. Kanter DR, Winiwarter W, Bodirsky BL, Bouwman L, Boyer E, Buckle S, et al. A framework for nitrogen futures in the shared socioeconomic pathways. Global Environmental Change 2020; 61: 102029.
13. Kc S, Lutz W. Demographic scenarios by age, sex and education corresponding to the SSP narratives. Population and Environment 2014; 35: 243-260.
14. Klein D, Luderer G, Kriegler E, Strefler J, Bauer N, Leimbach M, et al. The value of bioenergy in low stabilization scenarios: an assessment using REMIND-MAgPIE. Climatic change 2014; 123: 705-718.
15. Kriegler E, Bauer N, Popp A, Humpenöder F, Leimbach M, Strefler J, et al. Fossil-fueled development (SSP5): an energy and resource intensive scenario for the 21st century. Global environmental change 2017; 42: 297-315.
16. Lassaletta L, Billen G, Grizzetti B, Anglade J, Garnier J. 50 year trends in nitrogen use efficiency of world cropping systems: the relationship between yield and nitrogen input to cropland. Environmental Research Letters 2014; 9: 105011.
17. Li Y, Wang M, Chen X, Cui S, Hofstra N, Kroeze C, et al. Multi-pollutant assessment of river pollution from livestock production worldwide. Water Research 2022; 209: 117906.
18. O’Neill BC, Kriegler E, Ebi KL, Kemp-Benedict E, Riahi K, Rothman DS, et al. The roads ahead: Narratives for shared socioeconomic pathways describing world futures in the 21st century. Global Environmental Change 2017; 42: 169-180.
19. O’Neill BC, Kriegler E, Riahi K, Ebi KL, Hallegatte S, Carter TR, et al. A new scenario framework for climate change research: the concept of shared socioeconomic pathways. Climatic Change 2014; 122: 387-400.
20. Pastor A, Ludwig F, Biemans H, Hoff H, Kabat P. Accounting for environmental flow requirements in global water assessments. Hydrology and earth system sciences 2014; 18: 5041-5059.
21. Popp A, Dietrich JP, Lotze-Campen H, Klein D, Bauer N, Krause M, et al. The economic potential of bioenergy for climate change mitigation with special attention given to implications for the land system. Environmental Research Letters 2011; 6: 034017.
22. Schaphoff S, von Bloh W, Rammig A, Thonicke K, Biemans H, Forkel M, et al. LPJmL4–a dynamic global vegetation model with managed land–Part 1: Model description. Geoscientific Model Development 2018; 11: 1343-1375.
23. Smil V. Nitrogen in crop production: An account of global flows. Global biogeochemical cycles 1999; 13: 647-662.
24. Strokal M, Bai Z, Franssen W, Hofstra N, Koelmans AA, Ludwig F, et al. Urbanization: an increasing source of multiple pollutants to rivers in the 21st century. npj Urban sustainability 2021; 1: 1-13.
25. Strokal M, Ma L, Bai Z, Luan S, Kroeze C, Oenema O, et al. Alarming nutrient pollution of Chinese rivers as a result of agricultural transitions. Environmental Research Letters 2016; 11: 024014.
26. Strokal M, Spanier JE, Kroeze C, Koelmans AA, Flörke M, Franssen W, et al. Global multi-pollutant modelling of water quality: scientific challenges and future directions. Current Opinion in Environmental Sustainability 2019; 36: 116-125.
27. Swaney DP, Howarth RW, Hong B. Nitrogen use efficiency and crop production: Patterns of regional variation in the United States, 1987–2012. Science of the Total Environment 2018; 635: 498-511.
28. Tian H, Bian Z, Shi H, Qin X, Pan N, Lu C, et al. History of anthropogenic Nitrogen inputs (HaNi) to the terrestrial biosphere: a 5 arcmin resolution annual dataset from 1860 to 2019. Earth System Science Data 2022; 14: 4551-4568.

29. van Vliet M, van Beek L, Eisner S, Flörke M, Wada Y, Bierkens M. Multi-model assessment of global hydropower and cooling water discharge potential under climate change. Global Environmental Change 2016; 40: 156-170.
30. Van Vuuren DP, Edmonds J, Kainuma M, Riahi K, Thomson A, Hibbard K, et al. The representative concentration pathways: an overview. Climatic change 2011; 109: 5-31.
31. Van Vuuren DP, Stehfest E, Gernaat DE, Doelman JC, Van den Berg M, Harmsen M, et al. Energy, land-use and greenhouse gas emissions trajectories under a green growth paradigm. Global Environmental Change 2017; 42: 237-250.
32. Von Bloh W, Schaphoff S, Müller C, Rolinski S, Waha K, Zaehle S. Implementing the nitrogen cycle into the dynamic global vegetation, hydrology, and crop growth model LPJmL (version 5.0). Geoscientific Model Development 2018; 11: 2789-2812.
33. Wang M, Kroeze C, Strokal M, van Vliet MT, Ma L. Global change can make coastal eutrophication control in China more difficult. Earth's Future 2020a; 8: e2019EF001280.
34. Wang M, Ma L, Strokal M, Ma W, Liu X, Kroeze C. Hotspots for Nitrogen and Phosphorus Losses from Food Production in China: A County-Scale Analysis. Environmental Science & Technology 2018; 52: 5782-5791.
35. Wang Y, Xie Z, Liu S, Wang L, Li R, Chen S, et al. Effects of anthropogenic disturbances and climate change on riverine dissolved inorganic nitrogen transport. Journal of Advances in Modeling Earth Systems 2020b; 12: e2020MS002234.
36. Weindl I, Bodirsky BL, Rolinski S, Biewald A, Lotze-Campen H, Müller C, et al. Livestock production and the water challenge of future food supply: Implications of agricultural management and dietary choices. Global environmental change 2017a; 47: 121-132.
37. Weindl I, Popp A, Bodirsky BL, Rolinski S, Lotze-Campen H, Biewald A, et al. Livestock and human use of land: Productivity trends and dietary choices as drivers of future land and carbon dynamics. Global and Planetary Change 2017b; 159: 1-10.
38. Willett W, Rockström J, Loken B, Springmann M, Lang T, Vermeulen S, et al. Food in the Anthropocene: the EAT–Lancet Commission on healthy diets from sustainable food systems. The Lancet 2019; 393: 447-492.
39. Yu C, Huang X, Chen H, Godfray HCJ, Wright JS, Hall JW, et al. Managing nitrogen to restore water quality in China. Nature 2019; 567: 516-520.
40. Zhang X, Davidson EA, Mauzerall DL, Searchinger TD, Dumas P, Shen Y. Managing nitrogen for sustainable development. Nature 2015; 528: 51-59.
